# Design, synthesis, in vitro α-glucosidase inhibition, docking, and molecular dynamics of new phthalimide-benzenesulfonamide hybrids for targeting type 2 diabetes

**DOI:** 10.1038/s41598-022-14896-2

**Published:** 2022-06-22

**Authors:** Mohammad Askarzadeh, Homa Azizian, Mehdi Adib, Maryam Mohammadi-Khanaposhtani, Somayeh Mojtabavi, Mohammad Ali Faramarzi, Sayed Mahmoud Sajjadi-Jazi, Bagher Larijani, Haleh Hamedifar, Mohammad Mahdavi

**Affiliations:** 1 School of Chemistry, College of Science, University of Tehran, PO Box 14155-6455, Tehran, Iran; 2 Department of Medicinal Chemistry, School of Pharmacy, Iran University of Medical Sciences, Tehran, Iran; 3 Cellular and Molecular Biology Research Center, Health Research Institute, Babol University of Medical Sciences, Babol, Iran; 4 Department of Pharmaceutical Biotechnology, Faculty of Pharmacy, Tehran University of Medical Sciences, Tehran, Iran; 5 Endocrinology and Metabolism Research Center, Endocrinology and Metabolism Clinical Sciences Institute, Tehran University of Medical Sciences, Tehran, Iran; 6 Cell Therapy and Regenerative Medicine Research Center, Endocrinology and Metabolism Molecular-Cellular Sciences Institute, Tehran University of Medical Sciences, Tehran, Iran; 7 CinnaGen Medical Biotechnology Research Center, Alborz University of Medical Sciences, Karaj, Iran

**Keywords:** Biochemistry, Chemical biology, Computational biology and bioinformatics, Drug discovery

## Abstract

In the present work, a new series of 14 novel phthalimide-benzenesulfonamide derivatives **4a**–**n** were synthesized, and their inhibitory activity against yeast α-glucosidase was screened. The obtained results indicated that most of the newly synthesized compounds showed prominent inhibitory activity against α-glucosidase. Among them, 4-phenylpiperazin derivative **4m** exhibited the strongest inhibition with the IC_50_ value of 52.2 ± 0.1 µM. Enzyme kinetic study of compound **4m** proved that its inhibition mode was competitive and K_i_ value of this compound was calculated to be 52.7 µM. In silico induced fit docking and molecular dynamics studies were performed to further investigate the interaction, orientation, and conformation of the target compounds over the active site of α-glucosidase. Obtained date of these studies demonstrated that our new compounds interacted as well with the α-glucosidase active site with the acceptable binding energies. Furthermore, in silico druglikeness/ADME/Toxicity studies of compound **4m** were performed and predicted that this compound is druglikeness and has good ADME and toxicity profiles.

## Introduction

**Figure 1:**
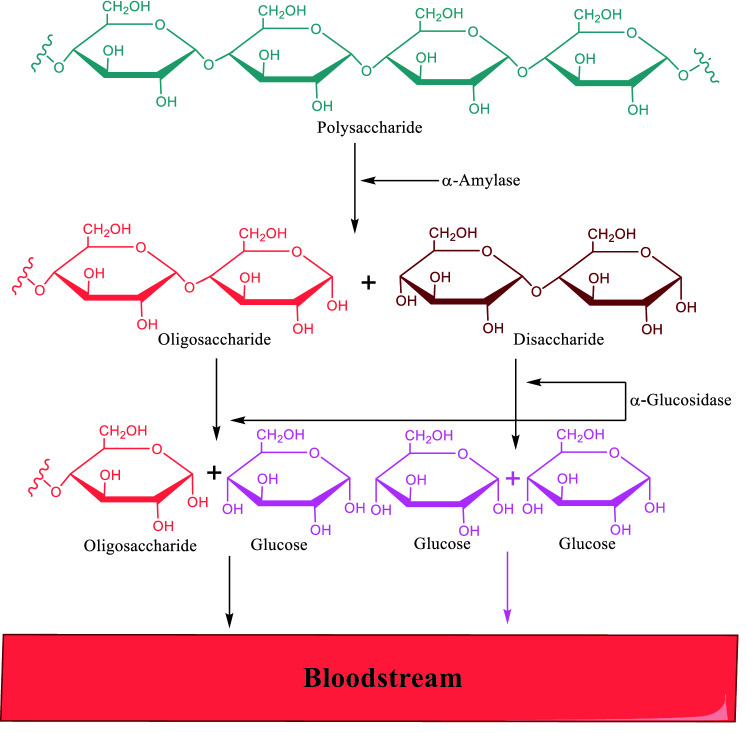
Schematic diagram illustrating the mechanism of action of α-amylase and α-glucosidase (ChemDraw 18.2).

Diabetes mellitus (DM) is a group of metabolic disorders that cause abnormally high blood glucose levels^1^. These disorders are mainly classified based on the role of insulin in them as the main hormone in regulating blood glucose^2^. In the insulin-dependent type (type 1), there are disorders in the insulin secretory cells, and in the non-insulin-dependent type (type 2), the body resists against the effect of insulin^3^. The most common type of DM is type 2 that associated with postprandial hyperglycemia. Commonly, postprandial hyperglycemia is markedly increased in diabetic patients with fasting hyperglycemia^4^. Therefore, the most important concern in regulating blood glucose in these patients is postprandial hyperglycemia regulation^5^. After eating, carbohydrates in food were hydrolyzed into glucose by two main groups of enzymes: α-amylase in the pancreas that converts polysaccharides to oligosaccharides and disaccharides and α-glucosidase in the intestine that converts oligosaccharides and disaccharides to glucose (Fig. 1)^6^. Among these two enzymes, inhibition of α-glucosidase has particular importance because inhibition of α-amylase led to an increase in section of undigested starch into the large intestine and gastrointestinal complications^7^. It should be noted, most α-glucosidase inhibitors which are available today as diabetes medicine, can also inhibit α-amylase, and therefore specific α-glucosidase inhibitors are probably more tolerable^8^. On the other hand, the potent and safe α-glucosidase inhibitors can be useful in the treatment of obesity and other α-glucosidase related diseases^9–11^.

Phthalimide is an important chemical structure in the design of new bioactive compounds^12^. This scaffold having various pharmacological activities including anticonvulsant, anticancer, antimicrobial, antiviral, and antidiabetic properties^13–17^. Phthalimide scaffold also was applied in the design of several series of the synthetic α-glucosidase inhibitors^18–20^. On the other hand, one of the other pharmacophores that in the design of new potent α-glucosidase inhibitors was applied is benzenesulfonamide^19,21^. Therefore, in continuation of our ongoing research on introducing new hybrid compounds with significant inhibitory activity against α-glucosidase, herein, we designed and synthesized a new series of phthalimide-benzenesulfonamide hybrids **4a**–**n**. These compounds were evaluated against α-glucosidase in vitro and in silico. In vitro α-amylase inhibition assay of these compounds was also performed.

## Results and discussion

### Designing

**Figure 2:**
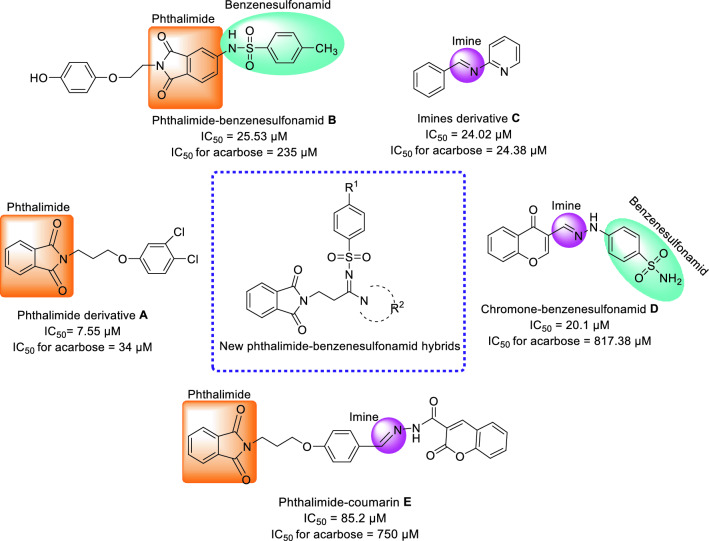
Design strategy for phthalimide-benzenesulfonamide hybrids based on scaffolds of the potent *α*-glucosidase inhibitors (**A**–**E**) (ChemDraw 18.2).

Phthalimide (isoindoline-1,3-dione) is a bicyclic heterocycle that applied as an important building block for design of the potent biological active compounds. As regards the derivatives of this scaffold such as compound **A** showed inhibitory activity against α-glucosidase, recently, use of it in designing new α-glucosidase inhibitors has received much attention (Fig. 2)^18^. For example, potent α-glucosidase inhibitor **B** was obtained by adding benzenesulfonamid, as another α-glucosidase inhibitor pharmacophor, to the phthalamide (Fig. 2)^19^.

Carbon–nitrogen double bond or imine group (C=N) is an important functional group in the design of the new bioactive compounds because it is an appropriate linker for connecting pharmacophores together. As regards the simple derivatives of imine such as compound **C** showed inhibitory activity against *α*-glucosidase, we selected imine as linker for connection of our selected pharmacophores (Fig. 2, compound **C** as the most potent compound)^22^. Two important samples of the strong α-glucosidase inhibitors that had imine group as linker in their structures, were chromone-benzenesulfonamid **D** (the most potent compound) and phthalimide-coumarin **E** (the most potent compound) that had been reported by our research group and Wang et al., respectively (Fig. 2)^20,21^.

Based on the mentioned points, herein, in view of scaffolds of **A**–**E** as the active compounds against α-glucosidase, we designed a new series of phthalimide-benzenesulfonamide hybrids and synthesized them by simple reactions. In vitro α-glucosidase inhibition assay of the all synthesized compounds, in vitro anti-α-amylase assay of the potent compounds against α-glucosidase, and kinetic analysis of the most potent entry were performed. In silico molecular modeling, molecular dynamics, and druglikeness/ADME/Toxicity profile of these compounds were also evaluated.

### Chemistry

**Scheme 1.**
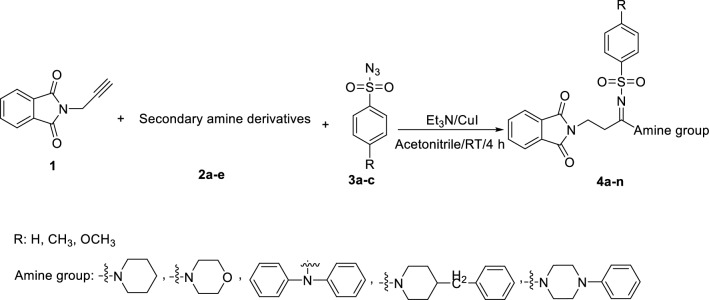
Synthesis of phthalimide-benzenesulfonamide derivatives **4a**–**n** (ChemDraw 18.2).

The synthetic route for the preparation of phthalimide-benzenesulfonamide derivatives **4a**–**n** has been depicted in Scheme 1. As can be seen in Scheme 1, *N*-propargylphthalimide **1** reacted with secondary amine derivatives **2a**–**e** and benzenesulfonyl azide derivatives **3a**–**c** in the presence of Et_3_N and CuI in acetonitrile to give target compounds **4a**–**n**.

### In vitro α-glucosidase inhibitory activity

All the synthesized analogues **4a**–**n** were evaluated for their in vitro anti-*α*-glucosidase activity. Acarbose was used as positive control and the results were summarized in Table 1. As can be seen in the Scheme 1, for the synthesis of target compounds, 5 type of secondary amines were used. On the other hand, there are phenyl, 4-methylphenyl, and or 4-methoxyphenyl groups in the benzenesulfonamide moiety. Enzymatic inhibition results revealed that most of the title compounds exhibited a convincingly potential *α*-glucosidase inhibition, in which compound **4m** bearing 4-phenylpiperazin and 4-methylphenyl ring was found as the most potent compound (IC_50_ = 52.2 ± 0.1 μM), which is nearly 14.5-fold more active than acarbose (IC_50_ = 750.0 ± 10.0 μM).

**Table 1:** In vitro* α*-glucosidase inhibitory activity of compounds **4a**–**n**.


Compound	Amine group	R	IC_50 (μM)_	Compound	Amine group	R	IC_50 (μM)_
**4a**		H	750 <	**4h**		CH_3_	497 ± 0.25
**4b**		CH_3_	127.24 ± 0.1	**4i**		H	84.3 ± 0.4
**4c**		OCH_3_	200.29 ± 0.1	**4j**		CH_3_	645.2 ± 0.08
**4d**		H	750 <	**4k**		OCH_3_	750 <
**4e**		CH_3_	117.03 ± 0.14	**4l**		H	750 <
**4f**		OCH_3_	750 <	**4m**		CH_3_	52.2 ± 0.1
**4g**		H	478 ± 0.07	**4n**		OCH_3_	750 <
Acarbose	–	–	–	–	–	–	750.0 ± 10.0

**Figure 3:**
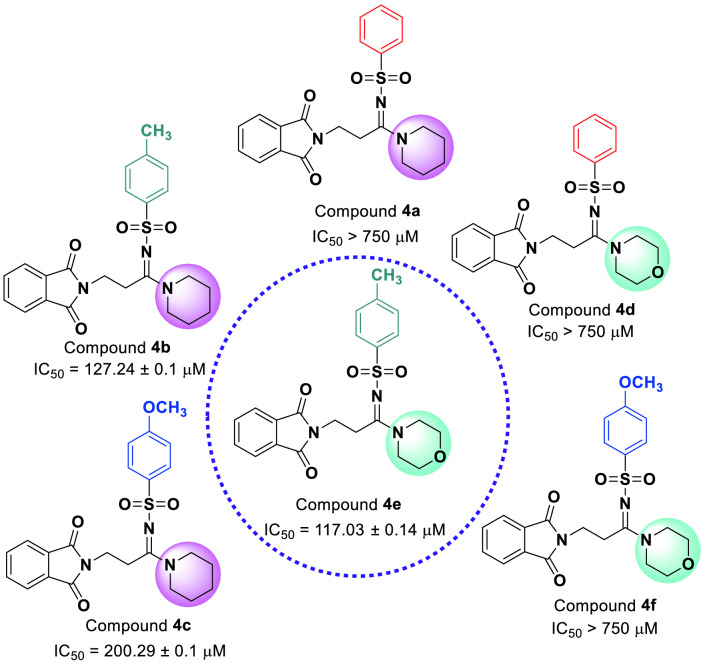
Comparison of α-glucosidase inhibitory activities of piperidine derivatives **4a**–**c** with morpholine derivatives **4d**–**f** (ChemDraw 18.2).

The comparison of IC_50_ values of piperidine derivatives **4a**–**c** with their corresponding morpholine derivatives **4d**–**f** against *α*-glucosidase revealed that phenyl derivatives (compounds **4a** and **4d**) in both group are inactive while 4-methylphenyl (compounds **4b** and **4e**) derivatives in both group showed high inhibitory activity (Fig. 3). In contrast, 4-methoxyphenyl derivative **4c** of piperidine series demonstrated a good anti-*α*-glucosidase activity while its corresponding morpholine analog was inactive.

**Figure 4:**
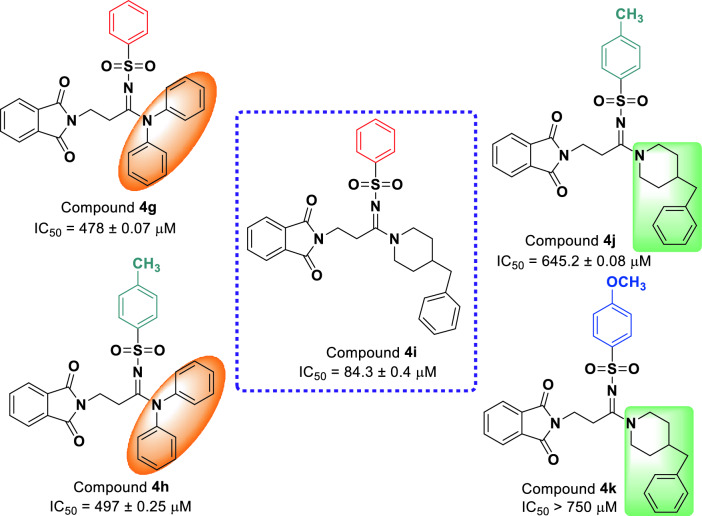
Comparison of α-glucosidase inhibitory activities of analogs **4g**–**k** (ChemDraw 18.2).

As can be seen in Fig. 4, phenyl derivative **4g** with *N*,*N*-diphenylamine moiety demonstrated moderate inhibitory activity while its corresponding analog with 4-benzylpiperidine **4i** showed high inhibitory activity against α-glucosidase. This compound was the second potent compound among the synthesized compounds. Both 4-methylphenyl analogs with *N*,*N*-diphenylamine and 4-benzylpiperidine, compounds **4h** and **4j**, were moderate α-glucosidase inhibitors. Moreover, 4-methoxyphenyl derivative **4k** with 4-benzylpiperidine moiety was inactive.

**Figure 5:**
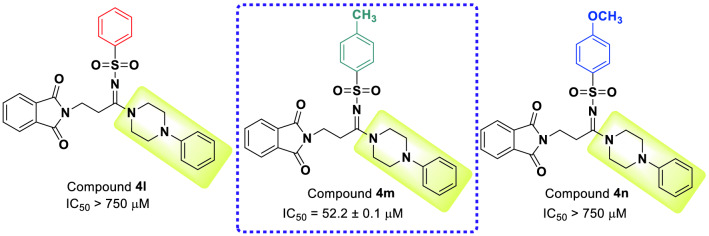
Comparison of α-glucosidase inhibitory activities of analogs **4l**–**n** (ChemDraw 18.2).

Finally, as can be seen in Fig. 5, among the 4-phenylpiperazin derivatives **4l**–**n**, only the compound **4m** containing 4-methyphenyl moiety was active. This compound was also the most potent compounds among the all synthesized compounds.

### Enzyme kinetic analysis

**Figure 6:**
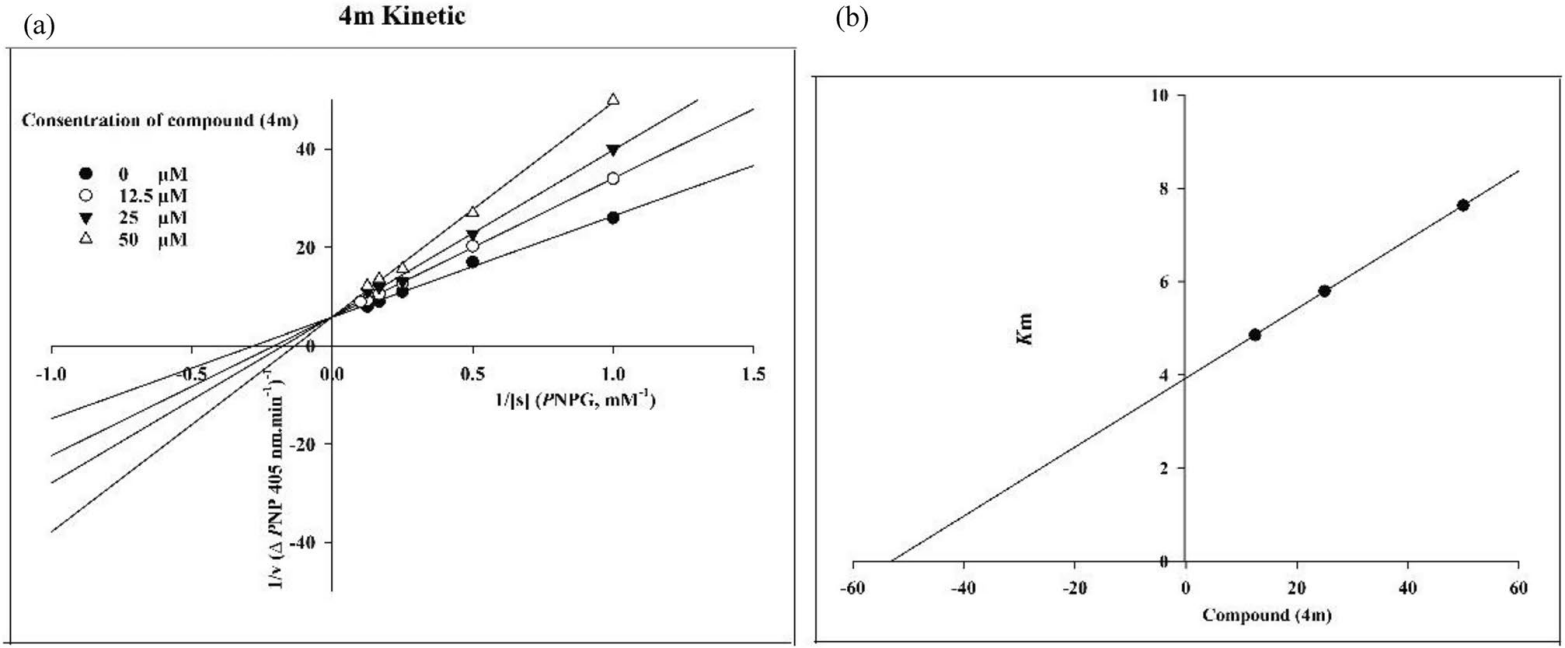
Kinetic study of the most potent inhibitor **4m** against α-glucosidase; (**a**) Lineweaver–Burk plot and (**b**) double reciprocal Lineweaver–Burk plot (Exel 2013).

Kinetic study of the most potent compound **4m** was performed by preparation of Lineweaver–Burk plot of this compound. The analysis of this plot demonstrated that with an increase in concentration of compound **4m**, V_max_ remains constant but the K_m_ increases. Therefore, compound **4m** competes with the substrate for binding to α-glucosidase (Fig. 6a). Moreover, K_i_ value of the latter compound was 52.7 μM that obtained by the plot of the K_m_ versus different concentrations of compound **4m** (Fig. 6b).

### Docking study

According to Yamamoto et al. study, the pocket shape of the active site α-glucosidase located at the interface of domain A and B, which contain conserve residues around the substrate-binding site^23^. Based on Davies et al. nomenclature, there are four subsides involved in substrate binding; − 1, + 1, + 2, and + 3 subsides^24^. The location and residue specification of α-glucosidase subsides are showed in Supplementary Fig. 1a. The − 1 and + 1 subsides consist of Asp214, Glu276, Asp349, Asp68, Tyr71, His111, Phe177, Gln181, and Arg348 (colored in green). The + 2 subside which are defined by Phe157, His239, Asn241 and Ala278 (colored in purple), whereas the + 3 subside depicted by His279, Glu304, Thr307 and Phe311 (colored in red)^25,26^.

The reliability of the applied docking protocol was assessed by re-docking of the enzyme substrate (α-D-glucose) into the active site of the α-glucosidase. Supplementary Fig. 1b shows the superimposed structures between the docked and the modeled α-D-glucose over α-glucosidase, which its RMSD is in acceptable value within the cutoff limit (0.32 Å).

Docking study was then applied to evaluate the interaction of newly synthetized compounds **4a**–**n** over the α-glucosidase active site and the obtained results were compared to acarbose. Table 2 shows docking score, glide score and IFD score of the top scoring pose of all the synthesized compounds. The obtained IFD scores are close to and correlated with the experimental results. Compounds **4m** and **4i** with the highest inhibitory activity represent the highest negative score of IFD − 1297.89 and − 1299.69 kcal/mol, respectively, while compounds **4a**, **4d**, **4f**, **4k**, **4l**, and **4n** with the lowest activities show lower negative score of IFD score (− 1293.25, − 1294.56, − 1294.02, − 1294.51, − 1295.92, and − 1294.98 kcal/mol, respectively).

**Table 2:** Docking score, glide score, and IFD score of compounds (**4a**–**4n**) over α-glycosidase.

Compound No	Docking score (Kcal/mol)	Glide energy (Kcal/mol)	IFD score (Kcal/mol)
**4a**	− 3.53	− 44.56	− 1293.25
**4b**	− 5.95	− 46.91	− 1295.91
**4c**	− 5.57	− 49.46	− 1294.61
**4d**	− 4.113	− 41.208	− 1294.56
**4e**	− 6.11	− 61.12	− 1294.77
**4f**	− 5.09	− 54.89	− 1294.02
**4g**	− 5.29	− 42.32	− 1295.05
**4h**	− 5.52	− 48.77	− 1295.87
**4i**	− 7.84	− 62.32	− 1299.69
**4j**	− 6.02	− 55.10	− 1296.01
**4k**	− 5.39	− 50.33	− 1294.51
**4l**	− 5.12	− 50.48	− 1295.92
**4m**	− 7.35	− 61.98	− 1297.89
**4n**	− 5.00	− 51.44	− 1294.98

**Figure 7:**
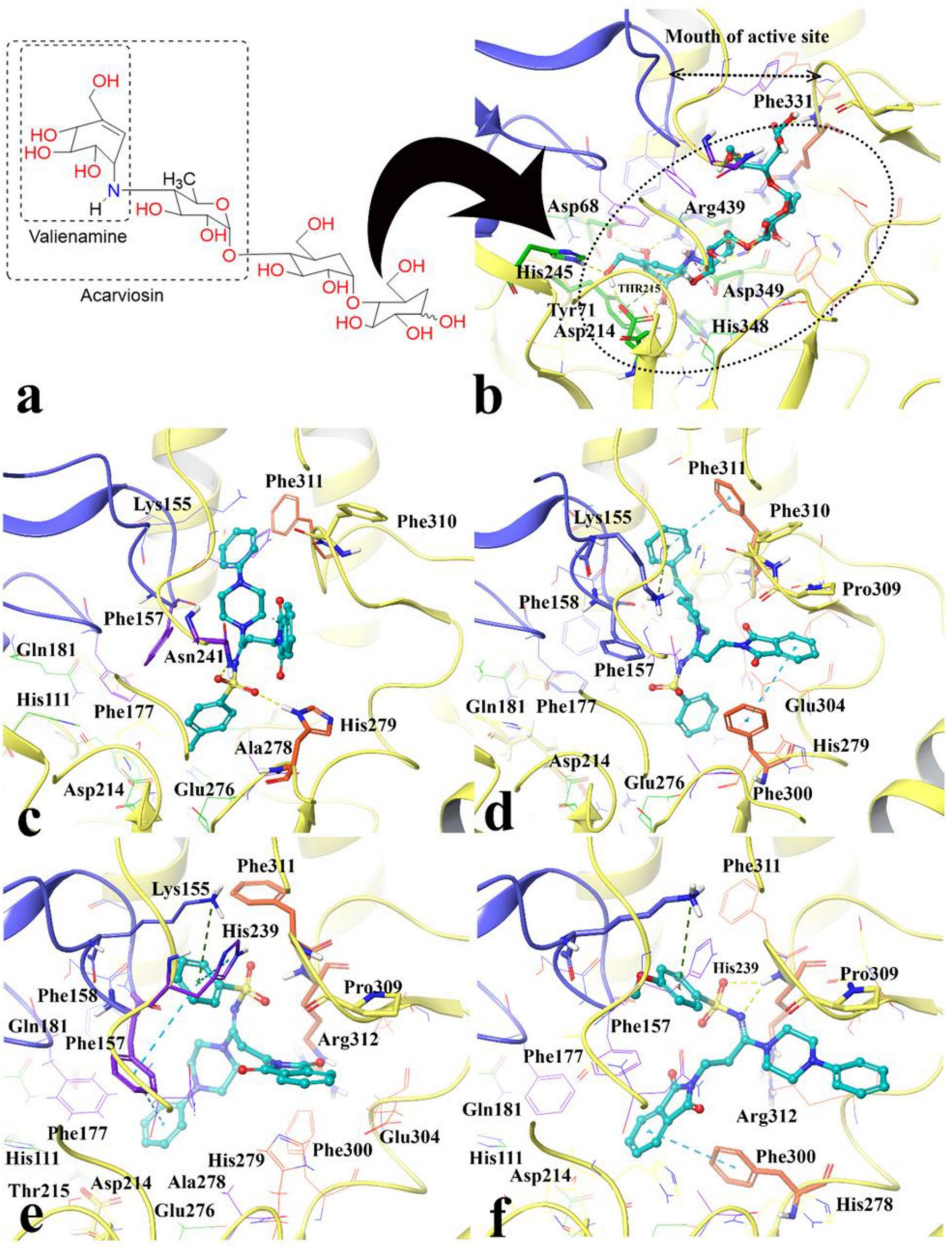
Acarbose structure (**a**) and docked representation of acarbose (**b**), the most active synthesized compounds **4m** (**c**) and **4i** (**d**) and the lowest active compounds **4l** (**e**) and **4n** (**f**) over the α-glucosidase active site. Domain A and B are colored in yellow and blue, respectively. The docked compounds colored in cyan. The α-glucosidase subsides residues include region − 1 and + 1 are in green color also the + 2 and + 3 subsides are in purple and orange, respectively (Maestro Molecular Modeling platform (version 12.5)).

The poses of the most active and the lowest active compounds were analyzed inside the binding site of α-glucosidase. Figure 7a and b show the structure and interaction mode of acarbose in which it deeply inserted into the active site. The valienamine moiety of acarbose which is corresponds to the non-reducing terminal of this compound interacted with Asp68, Tyr71, His111, Asp214, Asp349, His348 at the − 1 and + 1 subsides at the bottom of the active site. Furthermore, acarbose established hydrogen bonds with N–H units of Asn241 and Arg312 at the + 2 and + 3 subsides with distances 1.81 Å and 1.76 Å, respectively.

The molecular interactions of the best conformational pose and energy valued docked complex of compounds **4m** and **4i** with highest inhibition activity (Fig. 7c and d) and inactive compounds **4l** and **4n** (Fig. 7e and f) were also illustrated.

Like acarviosin moiety of acarbose, the 4-methylbenzensulfonamide moiety of compound **4m** pointed toward the − 1 subside which consists of the conserved catalytic residues Asp214, Asp349 and Glu276 and tightly stabilized by H-bond interactions with Asn241 and His279 belong to the + 2 and + 3 subsides (Fig. 7c). Furthermore, the phenylpiperazine group located between two loops at the hydrophobic entrance of the active site which are created by the residues at the tip of + 2 (Phe157) and + 3 (Phe311) subsides, respectively. In addition, phthalimide moiety positioned at the interface of the + 2 and + 3 subsides through hydrophobic interaction.

Among all compounds, compound **4i,** as the second potent compound, possessed the best docking score, glide energy and IFD score of − 7.84, − 62.32 kcal/mol, and − 1299.69, respectively. It depicts similar orientation and interaction to **4m** in which the phenylpiperazine group located between two loops at the hydrophobic entrance of the active site and stabilized through π-cation and π-π hydrophobic interactions with Lys155 from the + 2 subside and Phe311 from the + 3 subsides, respectively (Fig. 7d). The phthalimide moiety tends toward the + 3 subside in which it formed π-π hydrophobic interaction with Phe300 at the most bottom part. Also Fig. 7d reveals that the benzensulfonamide moiety oriented toward the − 1 subside while the phenyl group rotated toward the + 1 subside space.

On the other hand, compounds **4l** and **4n** with the lowest IFD score and inhibition activity showed different orientation with the potent compounds **4m** and **4i**. Benzensulfonamide moiety of both of compounds **4l** and **4n** totally shifted from the − 1 subside toward the active site mouth (Fig. 7e and f). The mentioned shifting observation may be as a result of the higher steric clash associated with the 4-methoxyphenyl moiety in compound **4n** and also the lack of the proper hydrophobic substituted at the mentioned position which observed in compound **4l**. In other word the para position of benzenesulfonamide moiety should provide the substitution with an optimum size in order to proper positioning toward the − 1 subside. This finding is consistence with our experimental inhibition assay in which compounds which has more steric 4-methoxy group (**4c**, **4f**, **4k**, and **4n**) or lack any proper hydrophobic group at the mentioned position (**4a**, **4d**, and **4l**) showed lower inhibition activity than acarbose.

Based on the observed results, it can be concluded that the proper positioning of benzensulfonamide moiety into the − 1 and + 1 subsides is important to reserve high enzyme inhibition activity as observed in compounds **4m** and **4i**.

By the way, in the case of compounds **4i**, **4j**, and **4k** with more bulky amine group like 4-benzylpiperidine, it can be observed that the lower size of the substitution over para benzenesulfonamide moiety is more favorable for high inhibitory action against α-glucosidase. The docking score, glide energy and IFD score of the mentioned compounds reveal that the energy related to the compound **4i** is more negative (− 7.84, − 62.32, and − 1299.69 kcal/mol) than **4j** (− 6.02, − 55.10, and − 1296.01), which is in accordance with the experimental data. So, the absence of only ‘–CH_3_ group’ intervene to achieving high inhibitory affinity for compound **4i** with more bulkier amine group, but presence of such group leading less inhibitory action against α-glucosidase for compound **4j**.

### Molecular dynamics investigation

The molecular dynamics (MD) simulation performed in order to understand the effect of the compound over the enzyme active site. For this purpose, the structural perturbations incurred by the most potent compound (compound **4m**) have been investigated over the active site environment.

**Figure 8:**
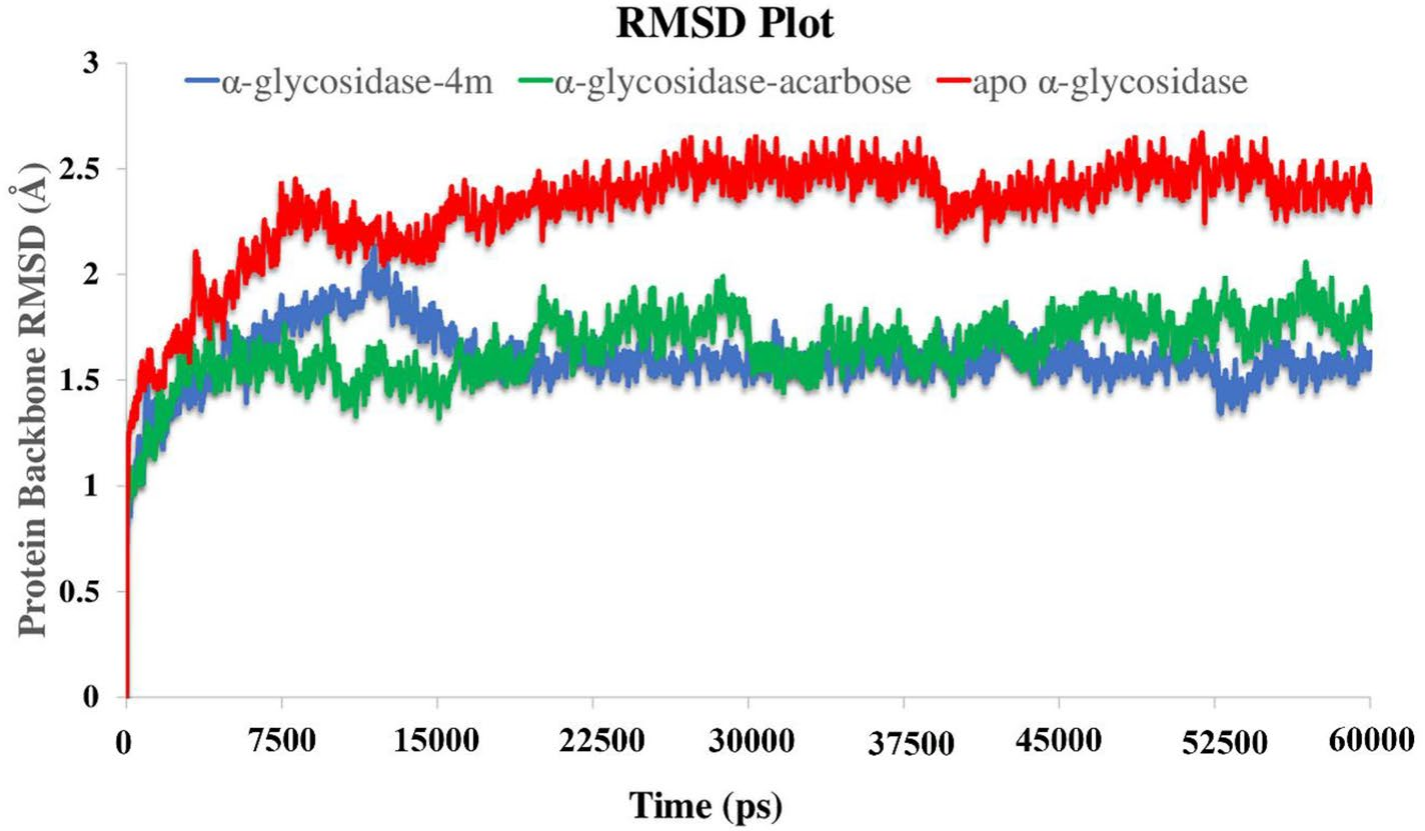
RMSD of the α-glucosidase backbone in complexed with acarbose (in green), compound **4m** (in blue) and the unbound enzyme (in red) for over 60 ns MD simulation time (Desmond v5.3).

Root mean square deviation (RMSD) of the α-glucosidase was analyzed over 60 ns MD simulation in order to evaluate the stability of the protein–ligand complex. The RMSD value of the unbounded target enzyme depicts higher RMSD value than the other two bounded-state enzyme complexes (Fig. 8). The RMSD value significantly increased during the first 7.5 ns up to 2.5 Å and steadily fluctuated to the next 30 ns and become more stable for the last 15 ns of the simulation time with the value of 2.6 Å (Fig. 8, red line). Moreover, based on the RMSD values of α-glucosidase-acarbose and α-glucosidase-compound **4m** complexes**,** the bounded-state enzymes were stable during the simulation time with the lower RMSD values (1.8 Å and 1.5 Å, respectively). The latter result indicated that the employed simulation time has been enough to obtain an equilibrium structure over the simulation time (Fig. 8, green and blue line).

**Figure 9:**
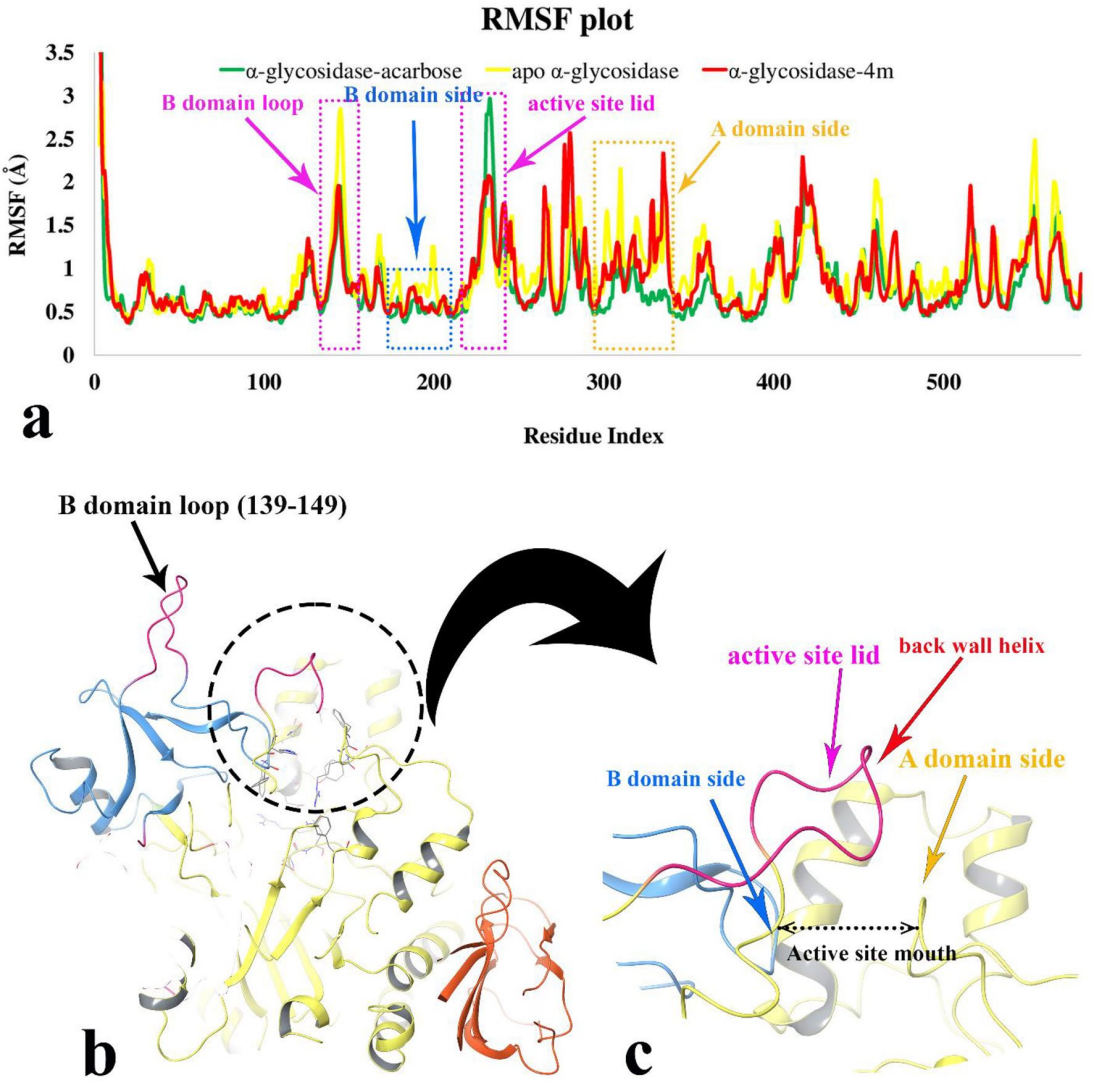
RMSF plot of the α-glycosidase backbone in complexed with compound **4m** (in red), acarbose (in green) and the apo enzyme (in yellow) for over 60 ns MD simulation time (**a**). 3D representation of α-glycosidase structure. Enzyme domain of A, B and C are colored in yellow, blue, and orange, respectively. The flexible regions correspond to B domain loop and active site lid are colored in pink (**b**). Close-up representation of α-glycosidase active site (**c**) (Desmond v5.3).

The RMSF plot which depicts the flexibility of protein structure showed that compound **4m** decreased the RMSF value of α-glycosidase residues in four regions as a result of non-bonding interactions; the B domain loop, the B domain side, the active site lid, and almost half of the A domain side (Fig. 9a). Furthermore, Fig. 9a showed that although the flexibility of the active site lid was the highest in enzyme acarbose bound-state, the mentioned segment flexibility revealed the lowest in α-glycosidase complexed with compound **4m**. Also, the organization of the α-glycosidase three main domains; A, B, and C along with the close-up representation of the active site mouth with the corresponding residues of A and B domains at the both sides of active site entrances represent in Fig. 9b and c, respectively. Based on the RMSF plot, acarbose and compound **4m** have almost the same interaction pattern through the whole α-glycosidase structure. The only dissimilarity comes from active site lid flexibility in which we can proposed that compound **4m** has superior effect in rigidity of active site lid rather than acarbose.

**Figure 10:**
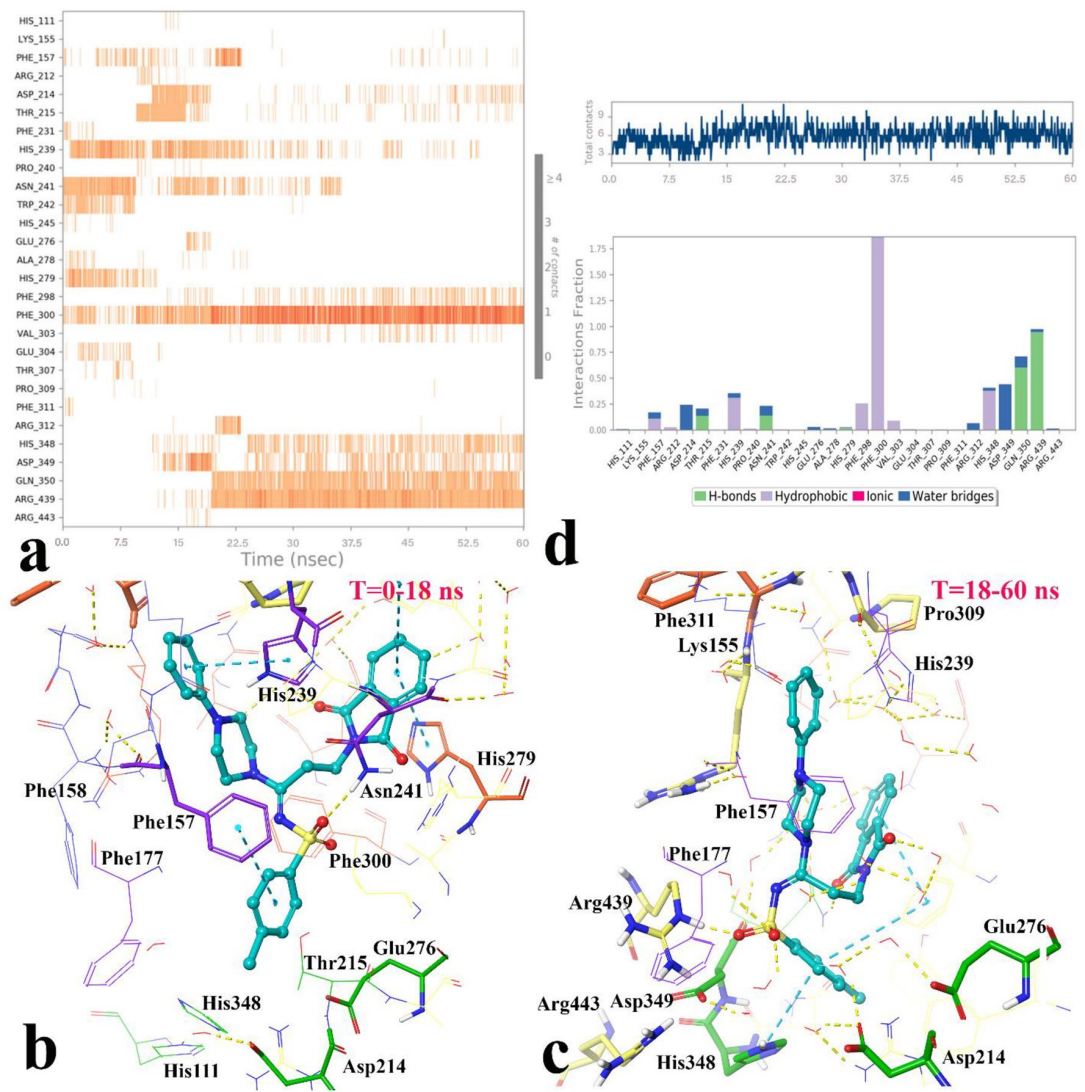
The timeline representation of the interactions shows the residues interact with compound **4m** in each trajectory frame (more than one specific contact with the ligand is represented by a darker shade of orange) (**a**). The 3D representation of α-glucosidase in bound-state with compound **4m** in two different orientations related to 0–15 ns (**b**) and 15 to the rest of simulation time (**c**). The quantity of contacts and the simulation interactions diagram panel in which the stacked bar charts are normalized over the course of the trajectory: some protein residues may make multiple contacts with the ligand (**d**) (Desmond v5.3).

In addition, different residues and types of interactions during the whole MD simulation time was showed in Fig. 10. Based on the timeline result, compound **4m** interacted with Phe157, His239, His241, and Ala278 which belong to the + 2 subside and Arg212, Trp242, His279, and Glu304 for approximate the first 15 ns of the MD simulation time. Otherwise, after about 15 ns until to the end of simulation some of interactions of the mentioned residues disappeared and substituted with other residues; Asp349, Asp214 which belong to the catalytic active site area, and Phe298, Phe300, His348, Gln350, Arg439 (Fig. 10a).

Based on the visual inspection of shifting orientation of compound **4m**, the MD simulation time divided in two sections; the first section was from 0 to 15 ns and the second one was from 15 to 60 ns. Based on the cluster analysis of compound **4m**, the percent of population in cluster 1 was 86.37% in the first section and 95% in the second section in which the representative frame from cluster 1 of the section 1 (Fig. 10b) and the section 2 (Fig. 10c) were selected for investigating the 3D complex interaction.

As it is obvious in Fig. 10b, at the first stage of MD simulation, compound **4m** coordinated in a way that the 4-methylbenzensulfonamide moiety oriented toward the − 1 subside. In addition, the 4-methylbenzensulfonamide, phenylpiperazine, and phthalimide aromatic rings provided π-π hydrophobic interactions with Phe157, His239 Asn241, and Trp242, respectively. Also, the sulfonamide moiety stabilized through H-bond interaction with Asn241 (2.3 Å). Otherwise, after about 15 ns the 4-methylbenzensulfonamide gradually rotated along its sigma C-N bond and flipped toward the + 3 subunit which was accompany with deep penetrating of the mentioned moiety toward the catalytic part of the active site, consequently electrophilic imine carbon (C = N) faced and exposed toward the three catalytic acidic residues; Asp214, Asp349, and Glu279. The new conformation and orientation of compound **4m** increased an average number of ligand-enzyme contacts from 4 to 8 interactions (Fig. 10d, up). In addition, Fig. 10a and c depict the sulfonamide moiety tightly stabilized with Arg439 through the strong H-bond interaction in 1.2 Å distance during a significant amount of the simulation time.

In addition to the interaction analysis, the Prime/MM-GBSA module was used to estimate the strengths of interactions between the ligand–protein complexes which generated by the clustering method. ΔG_bind_ of α-glycosidase/compound **4m** complex and α-glycosidase/acarbose complex were estimated to be − 92.13 and − 62.49 kcal/mol, respectively, revealing stronger binding interaction of compound **4m** than acarbose which also supported by experimental assay.

### In silico druglikeness/ADME/ toxicity studies

Druglikeness, ADME, and Toxicity profile of the most active compound **4m** and positive control acarbose were assigned using PreADMET as an online software and the obtained predictions were listed in Table 3. ^27^ As can be seen in Table 3, compound **4m** followed of Lipinski ‘Rule of five’ while acarbose did not follow of this rule. ADME prediction also showed that compound **4m** and acarbose have poor permeability to Caco-2 cell and their permeability to blood brain barrier (BBB) is in the normal range; compound **4m** has high human intestinal absorption (HIA) while acarbose did not have HIA. In term of toxicity, PreADMET predicted that compound **4m** and acarbose are both mutagenic; compound **4m** did not have carcinogenicity on mouse and rat while acarbose may have carcinogenicity on mouse. Furthermore, cardiotoxicity of acarbose is ambiguous while compound **4m** exhibited low risk in term of cardiotoxicity (hERG inhibition).

**Table 3:** Druglikeness/ADME/Toxicity prediction of the most active compound **4m** and acarbose.

Druglikeness/ADME/T ^a^	Compound	
	**4m**	Acarbose
Rule of Five	Suitable	Violated
Caco2	21.0452	9.44448
HIA	97.792576	0.000000
BBB	0.588671	0.0271005
Ames_test	Mutagen	Mutagen
Carcino_Mouse	Negative	Positive
Carcino_Rat	Negative	Negative
hERG inhibition	Low risk	Ambiguous

^a^The recommended ranges for Caco2: < 25 poor, > 500 great, HIA: > 80% is high < 25% is poor, and BBB = − 3.0 − 1.2.

### In vitro α-amylase assay

Among the new synthesized compounds, the eight active α-glucosidase inhibitors **4b**–**c**, **4e**, **4g**–**j**, and **4m** were selected for in vitro α-amylase inhibition assay^28^. Obtained results demonstrated that these compounds showed no inhibition effect against α-amylase at 300 μM while IC_50_ value of acarbose as positive control was 108 ± 0.71 μM. Therefore, our new active compounds against α-glucosidase considered as inactive compounds against α-amylase.

## Conclusion

In order to design of a new series of α-glucosidase inhibitors, herein, we used of molecular hybridization of two pharmacophores phthalimide and benzensulfonamid that are found in the potent α-glucosidase inhibitors. In this regards, 14 derivatives of the designed phthalimide-benzensulfonamid hybrids were synthesized and evaluated against α-glucosidase. Among the synthesized compounds, the most active compound **4m** (IC_50_ = 52.2 ± 0.1 μM) showed around 14.5 times better inhibitory activity than positive control, acarbose (IC_50_ = 750.0 ± 10.0 μM). Compound **4m** was a competitive inhibitor into α-glucosidase. IFD and MD studies showed that the proper positioning of benzensulfonamide moiety into the active site that is important to reserve high enzyme inhibition activity. In silico druglikeness/ADME/Toxicity profile of compound **4m** demonstrated that this compound is druglikeness and has the appropriate properties in terms of ADME and Toxicity. Furthermore, our new potent compounds against α-glucosidase were inactive against α-amylase.

## Experimental

### Methods

Melting points of the synthesized compounds **4a**–**n** were determined on a Kofler hot stage apparatus. ^1^H and ^13^C NMR spectra of title compounds were determined on a Bruker and Varian FT-500. Elemental analysis was obtained with an Elemental Analyzer system GmbH VarioEL CHN mode. Benzenesulfonyl azide derivatives **3a**–**c** were prepared according to the procedure described in the literature^29^.

### General procedure for the synthesis of dioxoisoindolin arylsulfonamides 4a–n

A mixture of *N*-propargylphthalimide **1** (1 mmol), secondary amine derivatives **2a**–**e** (1 mmol), benzenesulfonyl azide derivatives **3a**–**c** (1 mmol), Et_3_N (1.1 mmol), and CuI (10 mol %) in acetonitrile (2 mL) was stirred under N_2_ atmosphere for 1 h at room temperature. Then, mixture was stirred for 3 h at room temperature. After that, the reaction mixture was extracted with EtOAc (20 mL × 3). The organic phases were combined and solvent was evaporated under reduced pressure and the residue was purified directly by flash column chromatography (EtOAc/*n*hexane, 2:1) to afford the corresponding product **4a**–**n**.

#### ***N*-(3-(1,3-Dioxoisoindolin-2-yl)-1-(piperidin-1-yl)propylidene)benzenesulfonamide (4a)**

White solid, yield: 0.323 g (76%), m.p. = 158–160 °C. ^1^H NMR (500.1 MHz, CDCl_3_): *δ* = 1.55–1.65 (2H, m, CH_2_), 1.65–1.75 (4H, m, 2CH_2_), 3.33 (2H, t, *J* = 7.4 Hz, CH_2_), 3.65 and 3.68 (4H, 2br. t, *J* = 5.3 Hz, 2CH_2_), 3.96 (2H, t, *J* = 7.4 Hz, CH_2_), 7.37 (2H, t, *J* = 7.5 Hz, 2CH), 7.41 (1H, t, *J* = 7.5 Hz, CH), 7.67–7.69 (2H, m, 2CH), 7.78–7.80 (2H, m, 2CH), 7.85 (2H, d, *J* = 7.5 Hz, 2CH). ^13^C NMR (125.7 MHz, CDCl_3_): *δ* = 24.1, 25.2 and 26.7 (3CH_2_), 29.7 and 34.7 (N*C*H_2_*C*H_2_C), 46.2 and 47.8 (2CH_2_), 123.3 (2CH), 126.2 (2CH), 128.3 (2CH), 131.2 (CH), 132.0 (2C), 134.0 (2CH), 144.0 and 163.03 (2C), 167.84 (2C = O). Anal. Calcd for C_22_H_23_N_3_O_4_S: C, 62.10; H, 5.45; N, 9.88. Found: C, 62.19; H, 5.68; N, 10.07.

#### ***N*-(3-(1,3-Dioxoisoindolin-2-yl)-1-(piperidin-1-yl)propylidene)-4-methylbenzenesulfonamide (4b)**

White solid, yield: 0.298 g (68%), m.p. = 198–200 °C. ^1^H NMR (500.1 MHz, CDCl_3_): *δ* = 1.55–1.65 (2H, m, CH_2_), 1.65–1.75 (4H, m, 2CH_2_), 2.39 (3H, s, CH_3_), 3.37 (2H, t, *J* = 7.4 Hz CH_2_), 3.68 and 3.72 (4H, 2br. t, *J* = 5.5 Hz, 2CH_2_), 4.02 (2H, t, *J* = 7.4 Hz, CH_2_), 7.22 (2H, d, *J* = 7.9 Hz, 2CH), 7.73–7.75 (2H, m, 2CH), 7.79 (2H, d, *J* = 7.9 Hz, 2CH), 7.85–7.87 (2H, m, 2CH). ^13^C NMR (125.7 MHz, CDCl_3_): *δ* = 21.4 (CH_3_), 24.2, 25.2 and 26.8 (3CH_2_), 29.6 and 34.8 (N*C*H_2_*C*H_2_C), 46.2 and 47.8 (2CH_2_), 123.3 (2CH), 126.3 (2CH), 129.0 (2CH), 132.1 (2C), 134.0 (2CH), 141.2, 141.7 and 162.9 (3C), 167.9 (2C = O). Anal. Calcd for C_23_H_25_N_3_O_4_S: C, 62.85; H, 5.73; N, 9.56. Found: C, 62.71; H, 5.68; N, 9.73.

#### ***N*-(3-(1,3-dioxoisoindolin-2-yl)-1-(piperidin-1-yl)propylidene)-4-methoxybenzenesulfonamide (4c)**

White solid, yield: 0.336 g (74%), m.p. = 146–148 °C. ^1^H NMR (500.1 MHz, CDCl_3_): *δ* = 1.55–1.65 (2H, m, CH_2_), 1.65–1.75 (4H, m, 2CH_2_), 3.37 (2H, t, *J* = 7.5 Hz, CH_2_), 3.68 and 3.73 (4H, 2br. t, *J* = 5.1 Hz, 2CH_2_), 3.84 (3H, s, OCH_3_), 4.01 (2H, t, *J* = 7.5 Hz, CH_2_), 6.91 (2H, d, *J* = 7.3 Hz, 2CH), 7.72–7.75 (2H, m, 2CH), 7.86–7.88 (4H, m, 4CH). ^13^C NMR (125.7 MHz, CDCl_3_): *δ* = 24.2, 25.2 and 26.8 (3CH_2_), 29.5 and 34.8 (N*C*H_2_*C*H_2_C), 46.2 and 47.8 (2CH_2_), 55.5 (OCH_3_), 113.6 (2CH), 123.3 (2CH), 128.2 (2CH), 132.1 (2C), 134.0 (2CH), 136.2, 161.8 and 162.8 (3C), 167.8 (2C = O). Anal. Calcd for C_23_H_25_N_3_O_5_S: C, 60.64; H, 5.53; N, 9.22. Found: C, 60.52; H, 5.41; N, 9.38.

#### ***N*-(3-(1,3-dioxoisoindolin-2-yl)-1-morpholinopropylidene)benzenesulfonamide (4d)**

White solid, yield: 0.175 g (41%), m.p. = 168–170 °C. ^1^H NMR (500.1 MHz, CDCl_3_): *δ* = 3.36 (2H, t, *J* = 7.3 Hz, CH_2_), 3.68–3.80 (8H, m, 4CH_2_), 4.00 (2H, t, *J* = 7.3 Hz, CH_2_), 7.39 (2H, t, *J* = 7.4 Hz, 2CH), 7.44 (1H, t, *J* = 7.5 Hz, CH), 7.68–7.73 (2H, m, 2CH), 7.80–7.83 (2H, m, 2CH), 7.84 (2H, d, *J* = 7.4 Hz, 2CH). ^13^C NMR (125.7 MHz, CDCl_3_): *δ* = 29.4 and 34.7 (N*C*H_2_*C*H_2_C), 45.1 and 47.1 (2NCH_2_), 66.2 and 66.5 (2OCH_2_), 123.4 (2CH), 126.2 (2CH), 128.5 (2CH), 131.5 (CH), 132.0 (2C), 134.1 (2CH), 143.5 and 163.8 (2C), 167.9 (2C = O). Anal. Calcd for C_21_H_21_N_3_O_5_S: C, 59.00; H, 4.95; N, 9.83. Found: C, 58.89; H, 4.76; N, 9.96.

#### ***N*-(3-(1,3-Dioxoisoindolin-2-yl)-1-morpholinopropylidene)-4-methylbenzenesulfonamide (4e)**

White solid, yield: 0.273 g (62%), m.p. = 204–206 °C. ^1^H NMR (500.1 MHz, CDCl_3_): *δ* = 2.37 (3H, s, CH_3_), 3.36 (2H, t, *J* = 7.3 Hz, CH_2_), 3.70–3.85 (8H, m, 4CH_2_), 4.01 (2H, t, *J* = 7.3 Hz, CH_2_), 7.20 (2H, d, *J* = 7.9 Hz, 2CH), 7.70–7.78 (4H, m, 4CH), 7.83–7.85 (2H, m, 2CH). ^13^C NMR (125.7 MHz, CDCl_3_): *δ* = 21.4 (CH_3_), 29.3 and 34.7 (N*C*H_2_*C*H_2_C), 45.0 and 47.0 (2NCH_2_), 66.2 and 66.6 (2OCH_2_), 123.4 (2CH), 126.3 (2CH), 129.1 (2CH), 132.0 (2C), 134.1 (2CH), 140.7, 142.1 and 163.6 (3C), 167.9 (2C = O). Anal. Calcd for C_22_H_23_N_3_O_5_S: C, 59.85; H, 5.25; N, 9.52. Found: C, 59.74; H, 5.49; N, 9.66.

#### ***N*-(3-(1,3-Dioxoisoindolin-2-yl)-1-morpholinopropylidene)-4-methoxybenzenesulfonamide (4f)**

White solid, yield: 0.242 g (53%), m.p. = 172–174 °C. ^1^H NMR (500.1 MHz, CDCl_3_): *δ* = 3.36 (2H, t, *J* = 7.5 Hz, CH_2_), 3.70–3.79 (8H, m, 4CH_2_), 3.82 (3H, s, OCH_3_), 4.00 (2H, t, *J* = 7.5 Hz, CH_2_), 6.88 (2H, d, *J* = 8.8 Hz, 2CH), 7.71–7.74 (2H, m, 2CH), 7.79 (2H, d, *J* = 8.8 Hz, 2CH), 7.82–7.85 (2H, m, 2CH). ^13^C NMR (125.7 MHz, CDCl_3_): *δ* = 29.2 and 34.7 (N*C*H_2_*C*H_2_C), 45.0 and 47.0 (2NCH_2_), 55.5 (OCH_3_), 66.2 and 66.6 (2OCH_2_), 113.7 (2CH), 123.4 (2CH), 128.3 (2CH), 132.0 (2C), 134.1 (2CH), 135.6, 162.0 and 163.4 (3C), 167.9 (2C = O). Anal. Calcd for C_22_H_23_N_3_O_6_S: C, 57.76; H, 5.07; N, 9.18. Found: C, 57.84; H, 5.16; N, 9.25.

#### **3-(1,3-Dioxoisoindolin-2-yl)-N,N-diphenyl-N'-(phenylsulfonyl)propanimidamide (4g)**

White solid, yield: 0.417 g (82%), m.p. = 202–204 °C. ^1^H NMR (500.1 MHz, CDCl_3_): *δ* = 3.54 (2H, t, *J* = 6.1 Hz, CH_2_), 4.70 (2H, t, *J* = 6.1 Hz, CH_2_), 7.26–7.30 (12H, m, 12CH), 7.39 (1H, t, *J* = 7.4 Hz, CH), 7.57 (2H, d, *J* = 7.8 Hz, 2CH), 7.70–7.72 (2H, m, 2CH), 7.82–7.84 (2H, m, 2CH). ^13^C NMR (125.7 MHz, CDCl_3_): *δ* = 30.8 and 35.2 (N*C*H_2_*C*H_2_C), 123.2 (2CH), 126.1 (2CH), 127.2 (br.), 128.1 (2CH), 128.4 (br.), 129.0 (br.), 129.8 (br.), 131.2 (CH), 132.4 (2C), 133.8 (2CH), 143.1 (br., C − N), 165.7 (C), 168.0 (2 C = O). Anal. Calcd for C_29_H_23_N_3_O_4_S: C, 68.35; H, 4.55; N, 8.25. Found: C, 68.49; H, 4.71; N, 8.33.

#### **3-(1–3-Dioxoisoindolin-2-yl)-*N*,*N*-diphenyl-*N*’-tosylpropanimidamide (4h)**

White solid, yield: 0.475 g (91%), m.p. = 202–204 °C. ^1^H NMR (500.1 MHz, CDCl_3_): *δ* = 2.33 (3H, s, CH_3_), 3.54 (2H, t, *J* = 5.9 Hz, CH_2_), 4.05 (2H, t, *J* = 5.9 Hz, CH_2_), 7.06 (2H, d, *J* = 7.9 Hz, 2CH), 7.21–7.29 (10H, m, 10 CH), 7.44 (2H, d, *J* = 7.8 Hz, 2CH), 7.68–7.71 (2H, m, 2CH), 7.80–7.82 (2H, m, 2CH). ^13^C NMR (125.7 MHz, CDCl_3_): *δ* = 21.4 (CH_3_), 30.7 and 35.3 (N*C*H_2_*C*H_2_C), 123.2 (2CH), 126.1 (2CH), 127.2 (br.), 128.4 (br.), 128.7 (2CH), 129.0 (br.), 129.9 (br.), 132.3 (2C), 133.8 (2CH), 140.4 and 141.7 (2C), 141.9 (br., C − N), 143.6 (br., C − N), 165.5 (C), 168.02 (2C = O). Anal. Calcd for C_30_H_25_N_3_O_4_S: C, 68.82; H, 4.81; N, 8.03. Found: C, 68.75; H, 4.63; N, 8.17.

#### ***N*-(1-(4-benzylpiperidin-1-yl)-3-(1,3-dioxoisoindolin-2-yl)propylidene)benzenesulfonamide (4i)**

White solid, yield: 0.355 g (69%). ^1^H NMR (500.1 MHz, CDCl_3_): *δ* = 1.20–1.30 (2H, m, 2CH), 1.67 (1H, d, *J* = 13.5 Hz, CH), 1.80 (2H, d, *J* = 12.4 Hz, 2CH), 2.45–2.55 (2H, m, 2CH), 2.62 (1H, t, *J* = 13.1 Hz, CH), 3.08 (1H, t, *J* = 13.1 Hz, CH), 3.34 (2H, t, *J* = 7.4 Hz, CH_2_), 3.98 (2H, t, *J* = 7.3 Hz, CH_2_), 4.19 (1H, d, *J* = 13.2 Hz, CH), 4.72 (1H, d, *J* = 13.6 Hz, CH), 7.08 (2H, d, *J* = 7.4 Hz, 2CH), 7.17 (1H, t, *J* = 7.2 Hz, CH), 7.24 (2H, t, *J* = 7.4 Hz, 2CH), 7.38 (2H, t, *J* = 7.5 Hz, 2CH), 7.41 (1H, t, *J* = 7.0 Hz, CH), 7.66–7.68 (2H, m, 2CH), 7.78–7.79 (2H, m, 2CH), 7.87 (2H, d, *J* = 7.4 Hz, 2CH). ^13^C NMR (125.7 MHz, CDCl_3_): *δ* = 29.6 (NCH_2_*C*H_2_C), 31.3 and 32.7 (2CH_2_), 34.8 (N*C*H_2_CH_2_C), 37.7 (CH), 42.5, 45.4 and 47.0 (3CH_2_), 123.3 (2CH), 126.1 (CH), 126.2 (2CH), 128.3 (2CH), 128.5 (2CH), 129.0 (2CH), 131.3 (CH), 132.0 (2C), 134.1 (2CH), 139.6, 144.0 and 163.1 (3C), 167.8 (2C = O). Anal. Calcd for C_29_H_29_N_3_O_4_S: C, 67.55; H, 5.67; N, 8.15. Found: C, 67.39; H, 5.56; N, 8.23.

#### ***N*-(3-(1,3-dioxoisoindolin-2-yl)-1-(4-(4-methylbenzyl)piperidin-1-yl)propylidene)benzenesulfonamide (4j)**

White solid, yield: 0.381 g (73%). ^1^H NMR (500.1 MHz, CDCl_3_): *δ* = 1.20–1.30 (2H, m, 2CH), 1.69 (1H, d, *J* = 13.8 Hz, CH), 1.82 (2H, d, *J* = 12.4 Hz, 2CH), 2.35 (3H, s, CH_3_), 2.45–2.55 (2H, m, 2CH), 2.63 (1H, t, *J* = 13.0 Hz, CH), 3.09 (1H, t, *J* = 13.0 Hz, CH), 3.33 (2H, br. t, *J* = 7.3 Hz, CH_2_), 3.99 (2H, t, *J* = 7.3 Hz, CH_2_), 4.20 (1H, d, *J* = 13.4 Hz, CH), 4.75 (1H, d, *J* = 13.6 Hz, CH), 7.10 (2H, d, *J* = 7.5 Hz, 2CH), 7.15–7.25 (3H, m, 3CH), 7.27 (2H, t, *J* = 7.5 Hz, 2CH), 7.68–7.72 (2H, m, 2CH), 7.77 (2H, d, *J* = 7.5 Hz, 2CH), 7.80–7.84 (2H, m, 2CH). ^13^C NMR (125.7 MHz, CDCl_3_): *δ* = 21.4 (CH_3_), 29.6 (NCH_2_*C*H_2_C), 31.4 and 32.7 (2CH_2_), 34.8 (N*C*H_2_CH_2_C), 37.8 (CH), 42.5, 45.4 and 47.0 (3CH_2_), 123.3 (2CH), 126.1 (CH), 126.2 (2CH), 128.3 (2CH), 129.0 (2CH), 129.1 (2CH), 132.0 (2C), 134.1 (2CH), 139.6, 141.2, 141.7 and 163.0 (4C), 167.86 (2C = O). Anal. Calcd for C_30_H_31_N_3_O_4_S: C, 68.03; H, 5.90; N, 7.93. Found: C, 68.12; H, 5.83; N, 8.09.

#### ***N*-(3-(1,3-dioxoisoindolin-2-yl)-1-(4-(4-methoxybenzyl)piperidin-1-yl)propylidene)benzenesulfonamide (4k)**

White solid, yield: 0.430 g (79%). ^1^H NMR (500.1 MHz, CDCl_3_): *δ* = 1.20–1.30 (2H, m, 2CH), 1.70 (1H, d, *J* = 13.6 Hz, CH), 1.82 (2H, d, *J* = 12.2 Hz, 2CH), 2.45–2.60 (2H, m, 2CH), 2.63 (1H, t, *J* = 12.9 Hz, CH), 3.09 (1H, t, *J* = 13.0 Hz, CH), 3.33 (2H, t, *J* = 7.2 Hz, CH_2_), 3.80 (3H, s, OCH_3_), 3.98 (2H, t, *J* = 7.5 Hz, CH_2_), 4.20 (1H, d, *J* = 13.6 Hz, CH), 4.76 (1H, d, *J* = 13.3 Hz, CH), 6.88 (2H, d, *J* = 8.9 Hz, 2CH), 7.10 (2H, d, *J* = 7.4 Hz, 2CH), 7.18 (1H, t, *J* = 7.5 Hz, CH), 7.27 (2H, t, *J* = 7.3 Hz, 2CH), 7.69–7.71 (2H, m, 3CH), 7.80–7.83 (4H, m, 4CH). ^13^C NMR (125.7 MHz, CDCl_3_): *δ* = 29.5 (NCH_2_*C*H_2_C), 31.4 and 32.7 (2CH_2_), 34.8 (N*C*H_2_CH_2_C), 37.8 (CH), 42.5, 45.4 and 47.0 (3CH_2_), 55.5 (OCH_3_), 113.6 (2CH), 123.3 (2CH), 126.1 (CH), 128.2 (2CH), 128.3 (2CH), 129.0 (2CH), 132.0 (2C), 134.1 (2CH), 136.2, 139.6, 161.8 and 162.9 (4C), 167.8 (2C = O). Anal. Calcd for C_30_H_31_N_3_O_5_S: C, 66.04; H, 5.73; N, 7.70. Found: C, 66.15; H, 5.86; N, 7.83.

#### ***N*-(3-(1,3-dioxoisoindolin-2-yl)-1-(4-phenylpiperazin-1-yl)propylidene)benzenesulfonamide (4l)**

White solid, yield: 0.19 g (38%), m.p. = 160–164 °C. ^1^H NMR (500.1 MHz, CDCl_3_): *δ* = 3.23 (2H, br. t, *J* = 5.2 Hz, CH_2_), 3.33 (2H, br. t, *J* = 5.1 Hz, CH_2_), 3.45 (2H, t, *J* = 7.6 Hz, CH_2_), 3.94 (4H, br. t, *J* = 5.2 Hz, 2CH_2_), 4.09 (2H, t, *J* = 7.5 Hz, CH_2_), 6.93–6.97 (3H, m, 3CH) 7.31 (2H, t, *J* = 7.6 Hz, 2CH), 7.46 (2H, d, *J* = 7.4 Hz, 2CH), 7.49 (1H, t, *J* = 7.0 Hz, CH), 7.74–7.76 (2H, m, 2CH), 7.88–7.89 (2H, m, 2CH), 7.94 (2H, d, *J* = 8.0 Hz, 2CH). ^13^C NMR (125.7 MHz, CDCl_3_): *δ* = 29.6 and 34.8 (N*C*H_2_*C*H_2_C), 44.7, 46.5, 48.8 and 49.8 (4CH_2_), 116.6 (2CH), 120.9 (CH), 123.4 (2CH), 126.3 (2CH), 128.5 (2CH), 129.3 (2CH), 131.5 (CH), 132.1 (2C), 134.1 (2CH), 143.1, 148.8 and 163.5 (3C), 167.9 (2C = O). Anal. Calcd for C_27_H_26_N_4_O_4_S: C, 64.53; H, 5.21; N, 11.15. Found: C, 64.66; H, 5.38; N, 11.25.

#### ***N*-(3-(1,3-dioxoisoindolin-2-yl)-1-(4-(p-tolyl)piperazin-1-yl)propylidene)benzenesulfonamide (4m)**

White solid, yield: 0.252 g (49%), m.p. = 202–204 °C. ^1^H NMR (500.1 MHz, CDCl_3_): *δ* = 2.41 (3H, s, CH_3_), 3.22 (2H, br. t, *J* = 5.2 Hz, CH_2_), 3.32 (2H, br. t, *J* = 5.2 Hz, CH_2_), 3.43 (2H, t, *J* = 6.2 Hz, CH_2_), 3.93(4H, t, *J* = 5.1 Hz, 2CH_2_), 4.08 (2H, t, *J* = 6.2 Hz, CH_2_), 6.92 (2H,d, *J* = 7.7 Hz, 2CH), 6.94 (1H, t, *J* = 7.3 Hz, CH), 7.25 (2H, d, *J* = 8.0 Hz, 2CH), 7.30 (2H, t, *J* = 7.3 Hz, 2CH), 7.74–7.76 (2H, m, 2CH), 7.81 (2H, d, *J* = 8.3 Hz, 2CH), 7.85–7.89 (2H, m, 2CH). ^13^C NMR (125.7 MHz, CDCl_3_): *δ* = 21.5 (CH_3_), 29.5 and 34.8 (N*C*H_2_*C*H_2_C), 44.6, 46.4, 48.7 and 49.8 (4CH_2_), 116.5 (2CH), 120.8 (CH), 123.4 (2CH), 126.4 (2CH), 129.1 (2CH), 129.3 (2CH), 132.0 (2C), 134.1 (2CH), 140.8, 142.0, 150.3 and 163.4 (4C), 167.9 (2C = O). Anal. Calcd for C_28_H_28_N_4_O_4_S: C, 65.10; H, 5.46; N, 10.85. Found: C, 65.21; H, 5.58; N, 10.71.

#### ***N*-(3-(1,3-dioxoisoindolin-2-yl)-1-(4-(4-methoxyphenyl)piperazin-1-yl)propylidene)benzenesulfonamide (4n)**

White solid, yield: 0.292 g (55%), m.p. = 158–160 °C. ^1^H NMR (500.1 MHz, CDCl_3_): *δ* = 3.20 (2H, br. t, *J* = 5.2 Hz, CH_2_), 3.30 (2H, br. t, *J* = 5.1 Hz, CH_2_), 3.41 (2H, t, *J* = 7.3 Hz, CH_2_), 3.82 (3H, s, OCH_3_), 3.91 (4H, br. t, *J* = 5.0 Hz, 2CH_2_), 4.03 (2H, t, *J* = 7.3 Hz, CH_2_), 6.89 (5H, m, 5CH), 7.27 (2H, t, *J* = 7.7 Hz, 2CH), 7.71–7.72 (2H, m, 2CH), 7.83–7.84 (4H, m, 4CH). ^13^C NMR (125.7 MHz, CDCl_3_): *δ* = 29.4 and 34.4 (N*C*H_2_*C*H_2_C), 44.6, 46.4, 48.6 and 49.7 (4CH_2_), 55.5 (OCH_3_), 113.7 (2CH), 116.4 (2CH), 120.6 (CH), 123.3 (2CH), 128.3 (2CH), 129.3 (2CH), 132.0 (2C), 134.1 (2CH), 135.9, 150.4, 162.0 and 163.4 (4C), 167.9 (2C = O). Anal. Calcd for C_28_H_28_N_4_O_5_S: C, 63.14; H, 5.30; N, 10.52. Found: C, 63.22; H, 5.18; N, 10.68.

### In vitro inhibition assays

In vitro α-glucosidase inhibition assay of target compounds **4a**–**n**, kinetic analysis of the most potent compound, and in vitro α-amylase inhibitory activity of the selected compounds were performed exactly according to our previous work^29^.

### Molecular modeling procedure

Maestro Molecular Modeling platform (version12.5) by Schrödinger, LLC was performed to uncover out the interactions mode of the best active structures over α-glycosidase enzyme^30^. The protein 3D structure was implemented according to our previous study as a result of homology modeled based on high structural identity and sequence similarity with α-glucosidase (α-1,4-glucosidase) from *S. cerevisiae* (PDB code 3A4A)^31^.

The 2D representation of the synthesized compounds were drawn in Marvin 15.10.12.0 program (http://www.chemaxon.com) and converted into pdb file^32^. The Protein Preparation Wizard and the LigPrep module were used to prepare protein and ligand structure properly^33,34^. The missing side chains of the proteins were filled using the Prime tool and missing residues were updated.

The accurate side‑chain and backbone flexibility during ligand binding at the active site of α-glycosidase enzyme were predicted by IFD method using Glide software (Schrödinger LLC 2018, USA)^35^. As the kinetic study revealed competitive type inhibition mechanism against enzyme, the *α*-glucosidase active site was used to generate the grid for IFD calculation. The maximum 20 poses with receptor and ligand van der waals radii of 0.7 and 0.5, respectively considered. Residues within 5 Å of the α-D-glucose at the active site were refined followed by side-chain optimization. Structures whose Prime energy is more than 30 kcal/mol are eliminated based on extra precious Glide docking.

### Molecular dynamics simulation

Molecular dynamics (MD) simulation of this study was performed by using the Desmond v5.3 module implemented in Maestro interface (from Schrödinger 2018‐4 suite)^36,37^. The appropriate pose for MD simulation procedure of the compounds was achieved by IFD method.

In order to build the system for MD simulation, the protein–ligand complexes were solvated with SPC explicit water molecules and placed in the center of an orthorhombic box of appropriate size in the Periodic Boundary Condition. Sufficient counter‐ions and a 0.15 M solution of NaCl were also utilized to neutralize the system and to simulate the real cellular ionic concentrations, respectively. The MD protocol involved minimization, pre-production, and finally production MD simulation steps. In the minimization procedure, the entire system was allowed to relax for 2500 steps by the steepest descent approach. Then the temperature of the system was raised from 0 to 300 K with a small force constant on the enzyme in order to restrict any drastic changes. MD simulations were performed via NPT (constant number of atoms, constant pressure i.e. 1.01325 bar and constant temperature i.e. 300 K) ensemble. The Nose‐Hoover chain method was used as the default thermostat with 1.0 ps interval and Martyna‐Tobias‐Klein as the default barostat with 2.0 ps interval by applying isotropic coupling style. Long‐range electrostatic forces were calculated based on Particle‐mesh‐based Ewald approach with the he cut‐off radius for columbic forces set to 9.0 Å. Finally, the system subjected to produce MD simulations for 60 ns for protein–ligand complex. During the simulation every 1000 ps of the actual frame was stored. The dynamics behavior and structural changes of the systems were analyzed by the calculation of the root mean square deviation (RMSD) and RMSF. Subsequently, the energy-minimized structure calculated from the equilibrated trajectory system was evaluated for investigation of each ligand–protein complex interaction.

### Prime MM-GBSA

The ligand binding energies (ΔG_Bind_) were calculated for compound **4m** and acarbose using Molecular mechanics/generalized born surface area (MM GBSA) modules (Schrödinger LLC 2018) based on the following equation;

$$ \Delta {\text{G}}_{{{\text{Bind}}}} = {\text{ E}}_{{{\text{Complex}}}} {-} \, \left[ {{\text{E}}_{{{\text{Receptor}}}} + {\text{ E}}_{{{\text{Ligand}}}} } \right] $$

where ΔG _Bind_ is the calculated relative free energy which includes both ligand and receptor strain energy. E_Complex_ is the MM-GBSA energy of the minimized complex, and E _Ligand_ is the MM-GBSA energy of the ligand after removing it from the complex and allowing it to relax. E _Receptor_ is the MM-GBSA energy of relaxed protein after separating it from the ligand. The MM-GBSA calculation was performed based on the clustering method for energy calculation^38,39^.

### In silico druglikeness/ADME/T prediction

In silico druglikeness/ADME/T studies of most avtive compound and positive control acarbose were carried out using by preADMET online server (http://preadmet.bmdrc.org/)^28^.

## Supplementary Information

Supplementary Information.
