# Supplementary material for: Design, synthesis, in vitro α-glucosidase inhibition, docking, and molecular dynamics of new phthalimide-benzenesulfonamide hybrids for targeting type 2 diabetes
Source: Sci Rep. 2022 Jun 22;12:10569. doi: 10.1038/s41598-022-14896-2 (PMC9217978; doi:10.1038/s41598-022-14896-2)
Supplement: Supplementary file 1 — Supplementary Information. [file 41598_2022_14896_MOESM1_ESM.docx]

**Support information**

**Design, synthesis, *in vitro* α-glucosidase inhibition, docking, and molecular dynamics of new phthalimide-benzenesulfonamide hybrids for targeting type 2 diabetes**

Mohammad Askarzadeh^1^, Homa Azizian^2^, Mehdi Adib^1^*, Maryam Mohammadi-Khanaposhtani^3^, Somayeh Mojtabavi^4^, Mohammad Ali Faramarzi^4^, Sayed Mahmoud Sajjadi-Jazi^5,6^, Bagher Larijani^5^, Haleh Hamedifar^7^, Mohammad Mahdavi^5^*

^1^School of Chemistry, College of Science, University of Tehran, PO Box 14155-6455, Tehran, Iran, ^✉^e-mail: [madib@khayam.ut.ac.ir](mailto:madib@khayam.ut.ac.ir) (M. Adib). ^2^Department of Medicinal Chemistry, School of Pharmacy, Iran University of Medical Sciences, Tehran, Iran. ^3^Cellular and Molecular Biology Research Center, Health Research Institute, Babol University of Medical Sciences, Babol, Iran. ^4^Department of Pharmaceutical Biotechnology, Faculty of Pharmacy, Tehran University of Medical Sciences, Tehran, Iran. ^5^Endocrinology and Metabolism Research Center, Endocrinology and Metabolism Clinical Sciences Institute, ^✉^e-mail: [momahdavi@tums.ac.ir](mailto:momahdavi@tums.ac.ir) (M. Mahdavi). Tehran University of Medical Sciences, Tehran, Iran. ^6^Cell Therapy and Regenerative Medicine Research Center, Endocrinology and Metabolism Molecular-Cellular Sciences Institute, Tehran University of Medical Sciences, Tehran, Iran. ^7^CinnaGen Medical Biotechnology Research Center, Alborz University of Medical Sciences, Karaj, Iran.


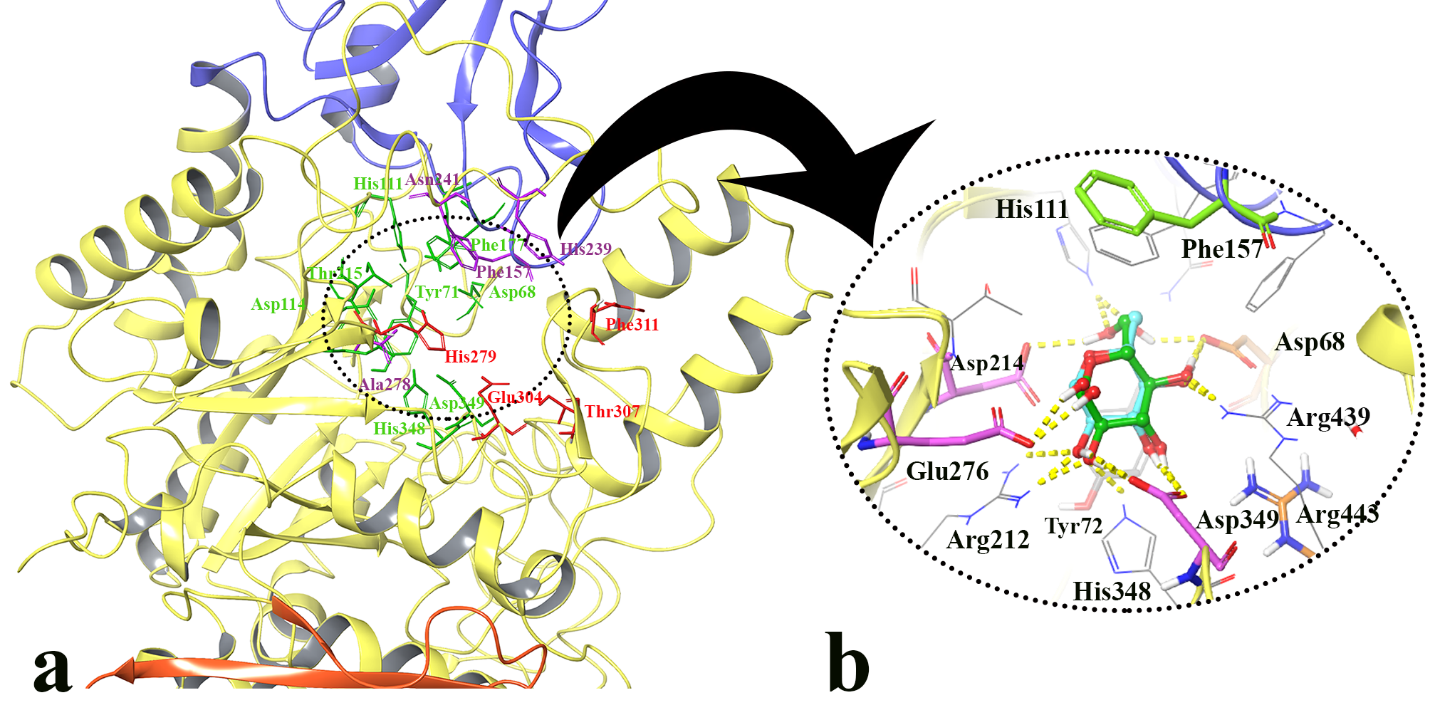


**Supplementary Fig. 1.** Representation of α-glucosidase active site. (a) The environment of α-glucosidase active site located at the interface of domain A and B. Domain A, B, and C are colored in yellow, blue, and red, respectively. In addition, the binding subunits are depicted as colored residues in which -1 and +1 subunits are in green color. The +2 and +3 subunits are in purple and red color, respectively (b) Close-up representation of active site, the modeled α-D-gloucose and the corresponding re-docked form represented in green and cyan color, respectively.

*N-(3-(1,3-dioxoisoindolin-2-yl)-1-(piperidin-1-yl)propylidene)benzenesulfonamide* ***4a*** *
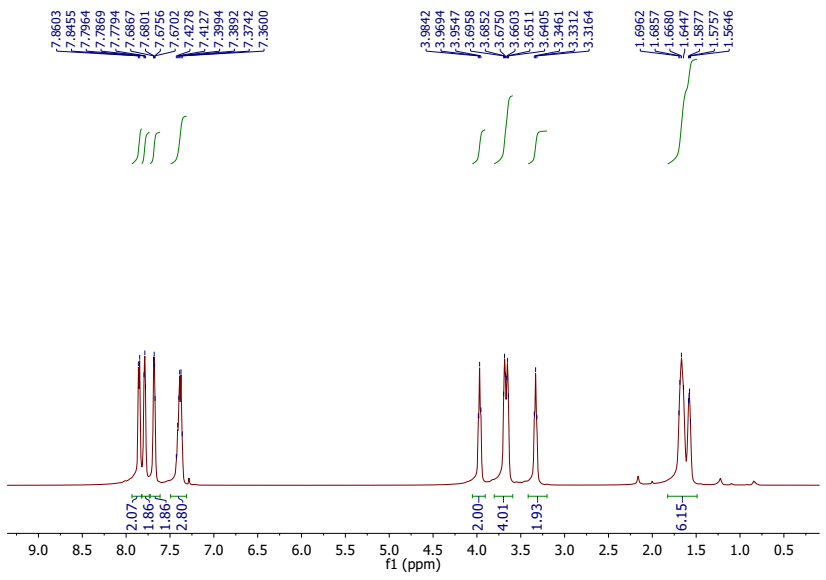

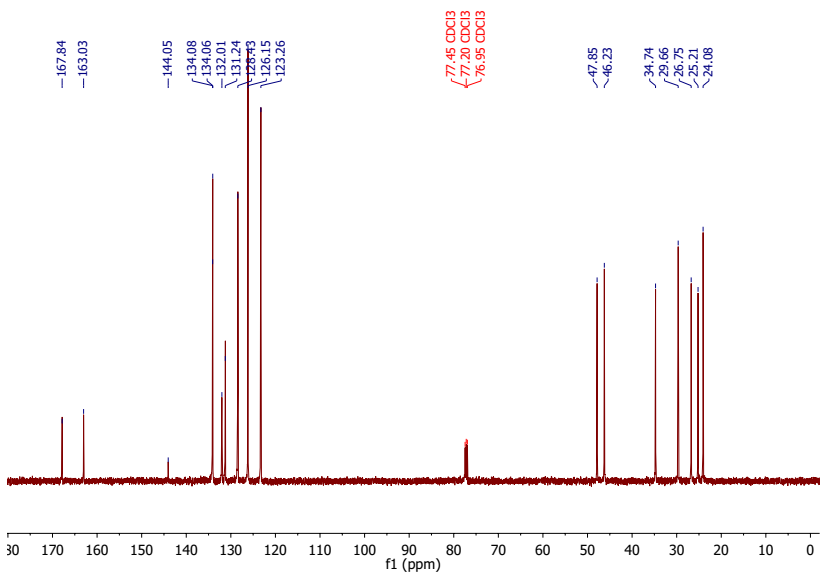
*

*N-(3-(1,3-dioxoisoindolin-2-yl)-1-(piperidin-1-yl)propylidene)-4-methylbenzenesulfonamide* ***4b***

*
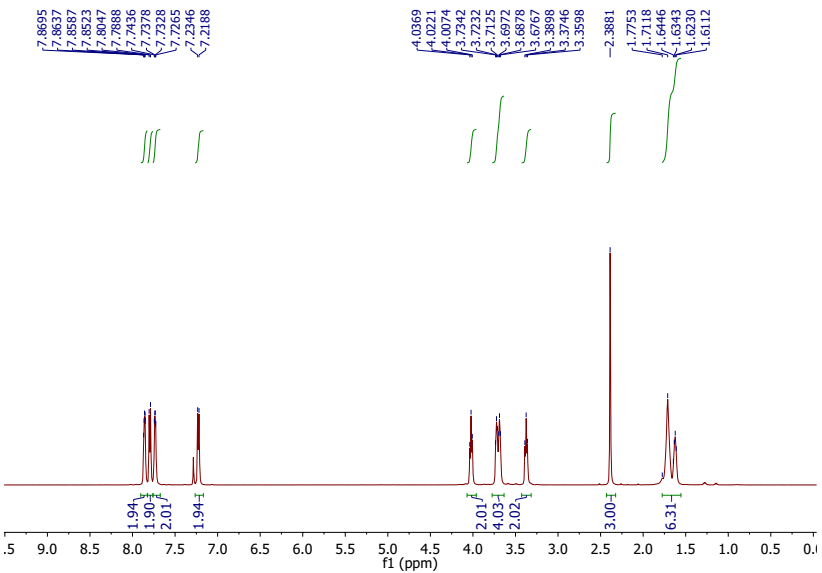

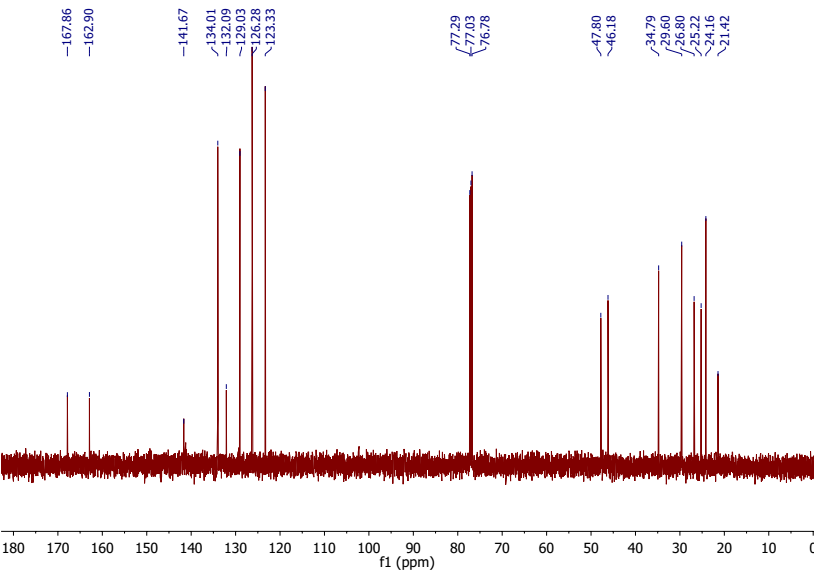
*

*N-(3-(1,3-dioxoisoindolin-2-yl)-1-(piperidin-1-yl)propylidene)-4-methoxybenzenesulfonamide* ***4c***

*
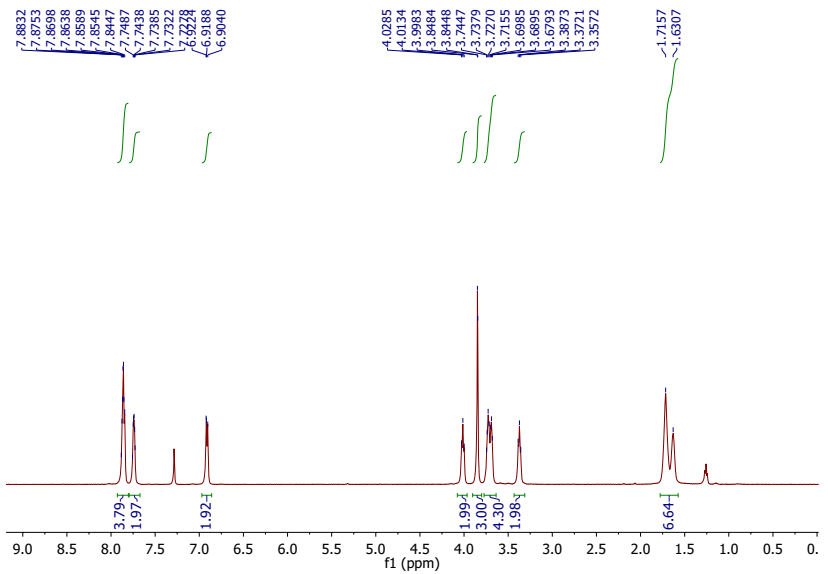

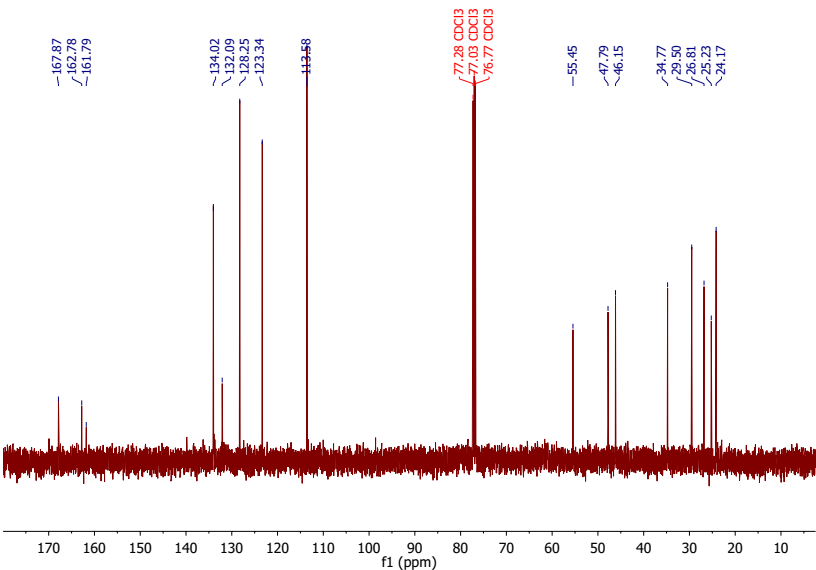
*

*N-(3-(1,3-dioxoisoindolin-2-yl)-1-morpholinopropylidene)benzenesulfonamide* ***4d*** *
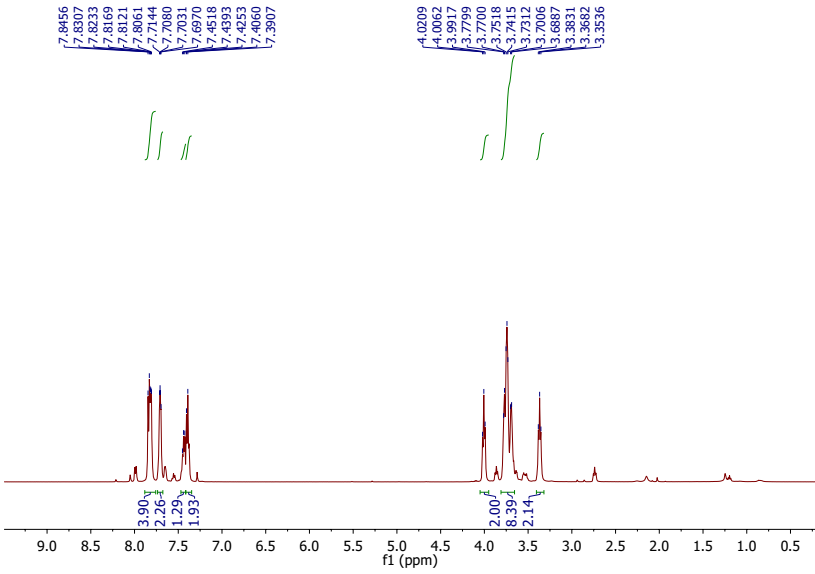

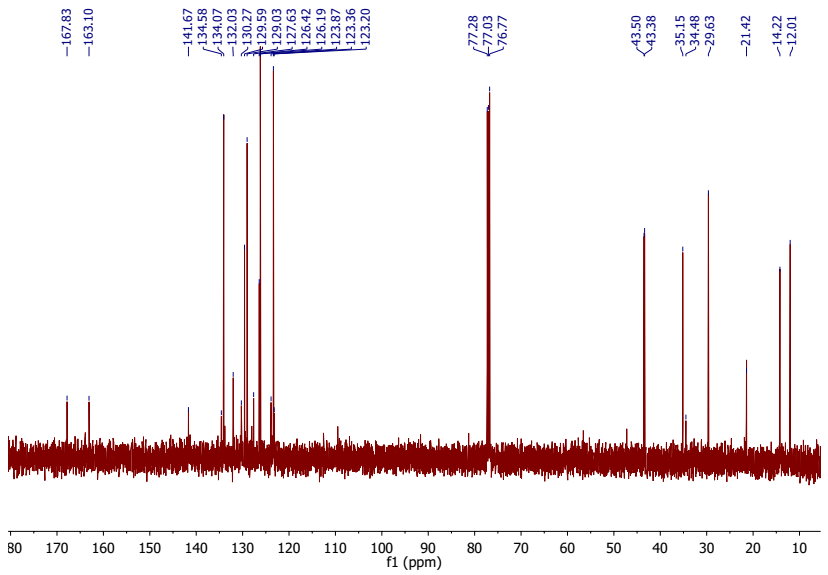
*

*N-(3-(1,3-dioxoisoindolin-2-yl)-1-morpholinopropylidene)-4-methylbenzenesulfonamide*

***4e***

*
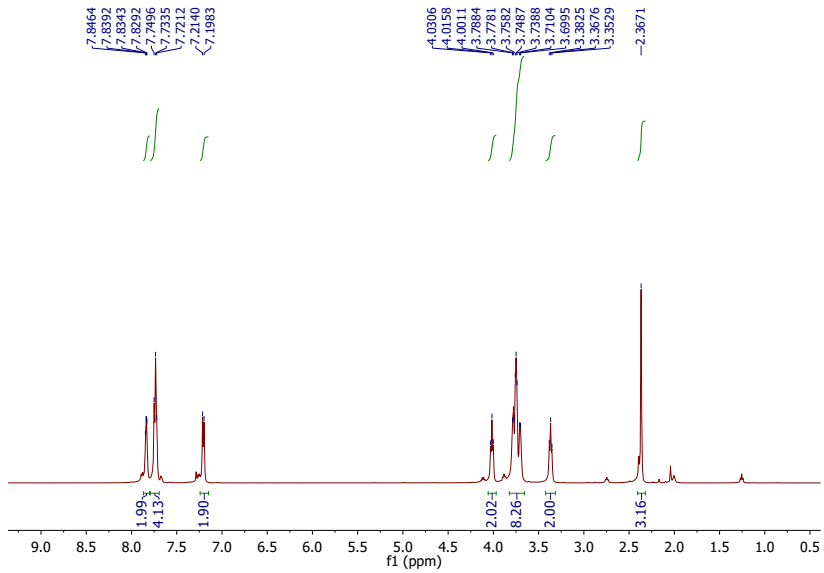

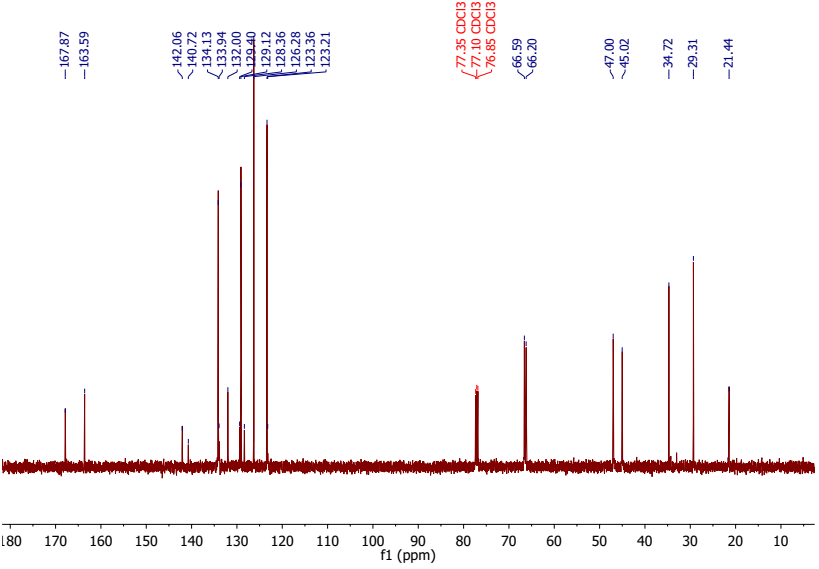
*

*N-(3-(1,3-dioxoisoindolin-2-yl)-1-morpholinopropylidene)-4-methoxybenzenesulfonamide*

***4f***

*
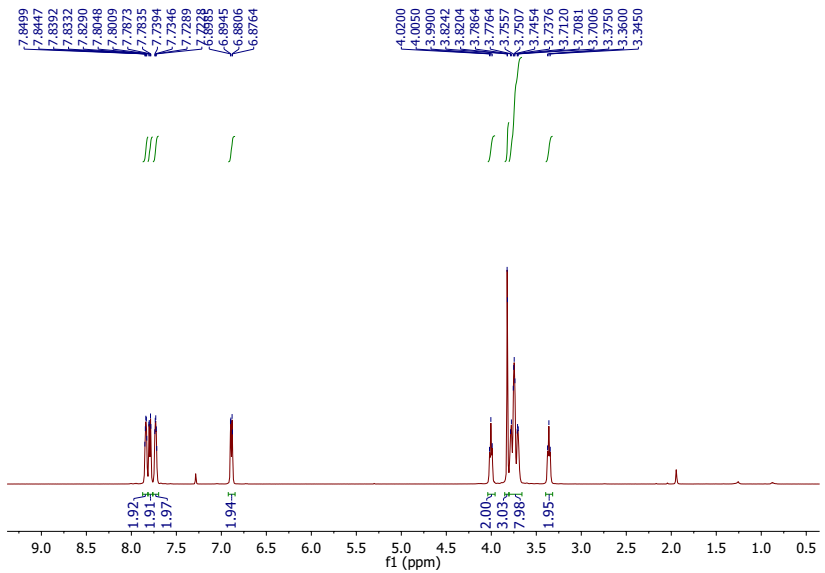

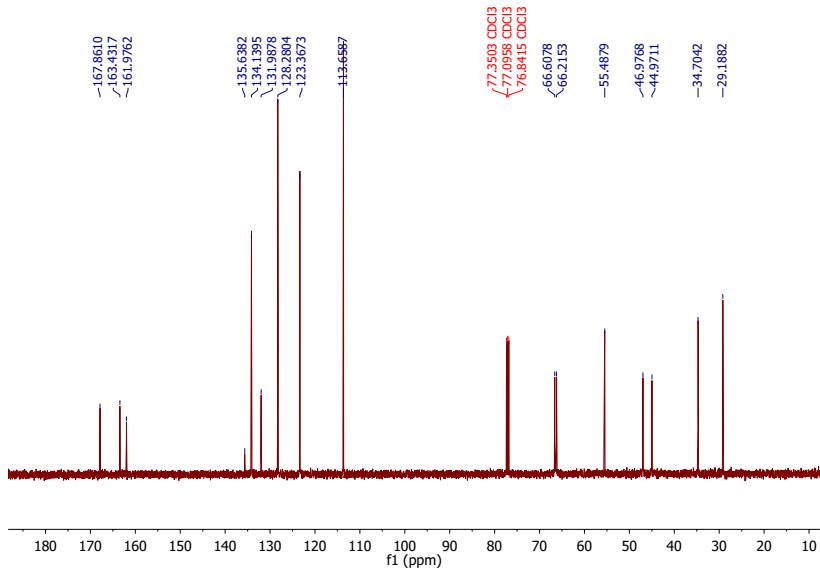
*

*3-(1,3-dioxoisoindolin-2-yl)-N,N-diphenyl-N'-(phenylsulfonyl)propanimidamide* ***4g*** *
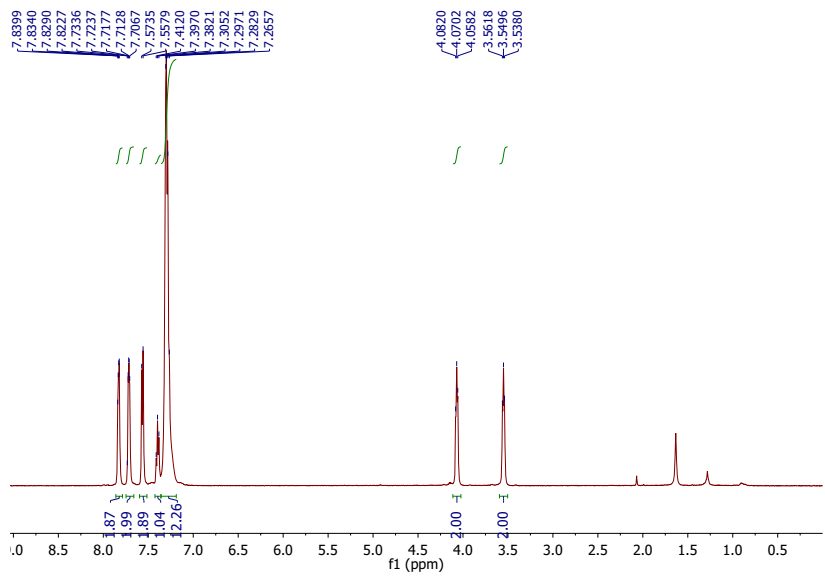

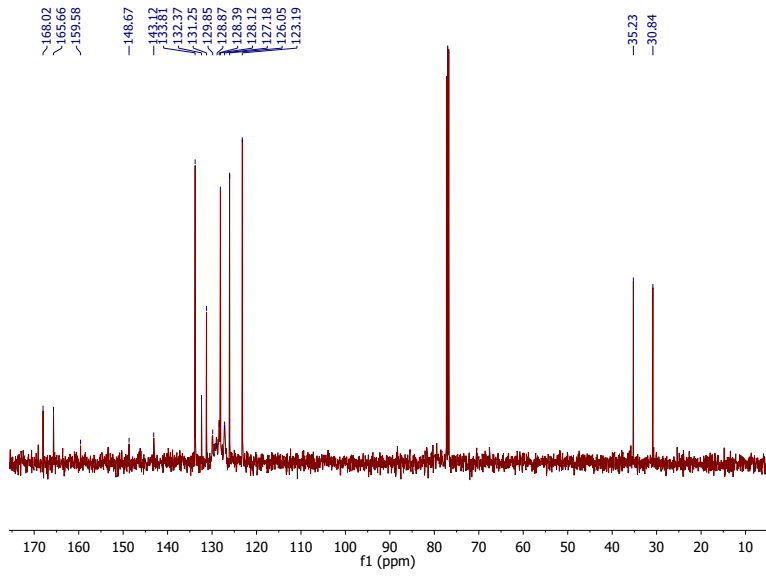
*

*3-(1-3-dioxoisoindolin-2-yl)-N,N-diphenyl-N’-tosylpropanimidamide* ***4h*** *
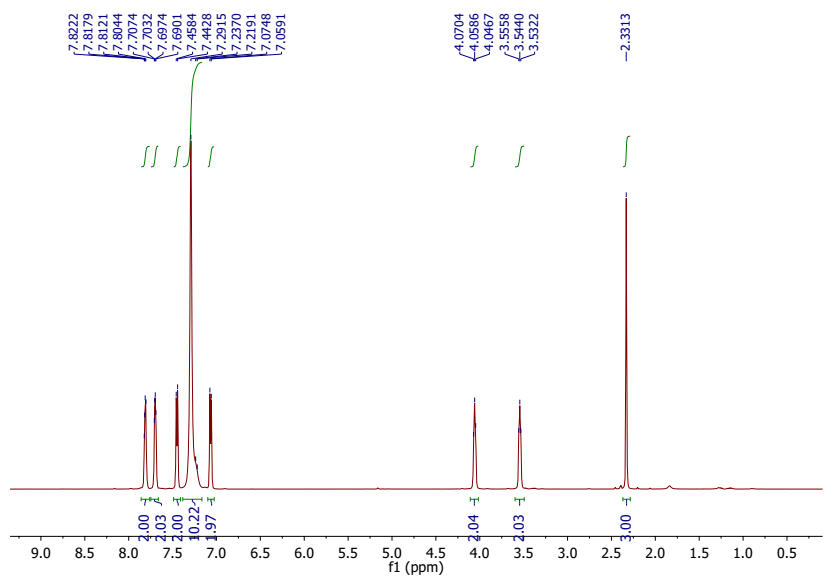

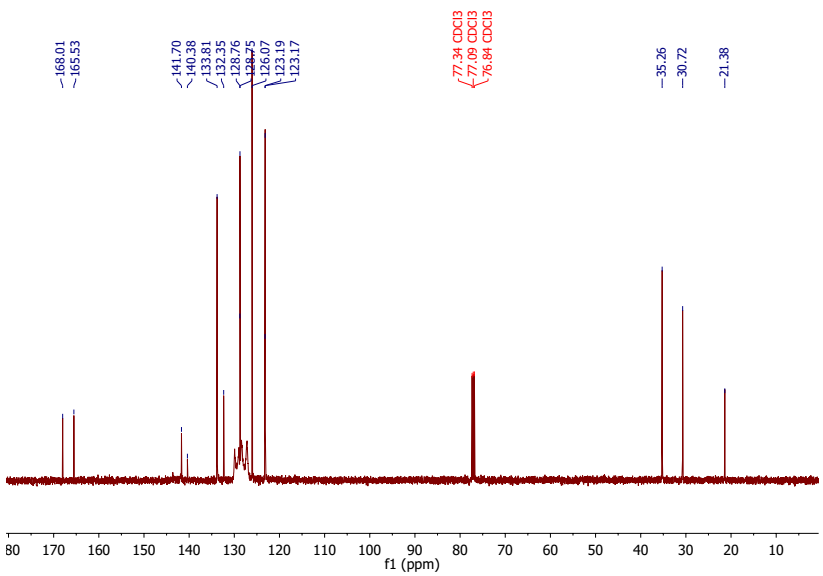
*

*N-(1-(4-benzylpiperidin-1-yl)-3-(1,3-dioxoisoindolin-2-yl)propylidene)benzenesulfonamide* ***4i***

*
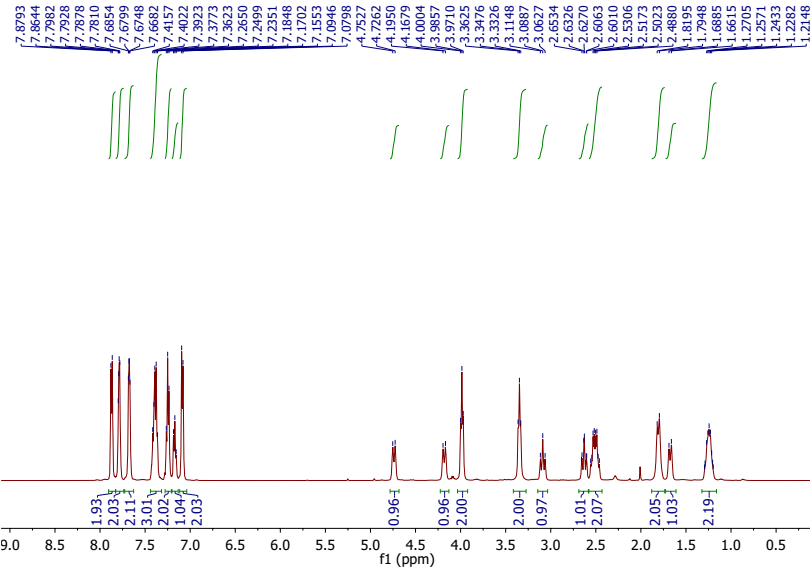

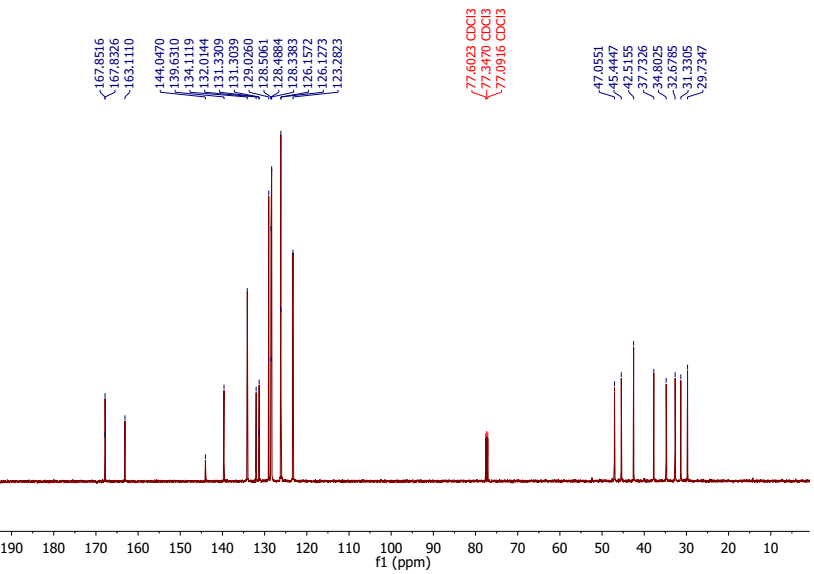
*

*N-(3-(1,3-dioxoisoindolin-2-yl)-1-(4-(4-methylbenzyl)piperidin-1-yl)propylidene)benzenesulfonamide* ***4j***

*
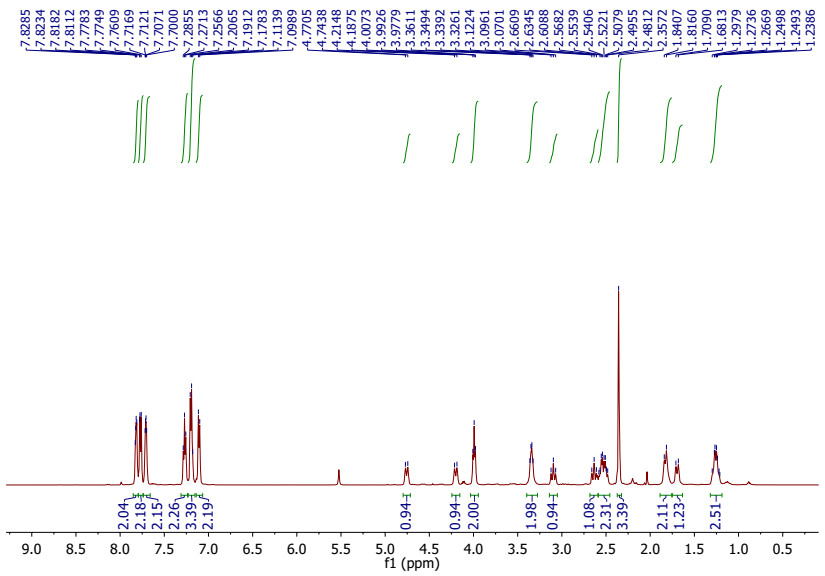

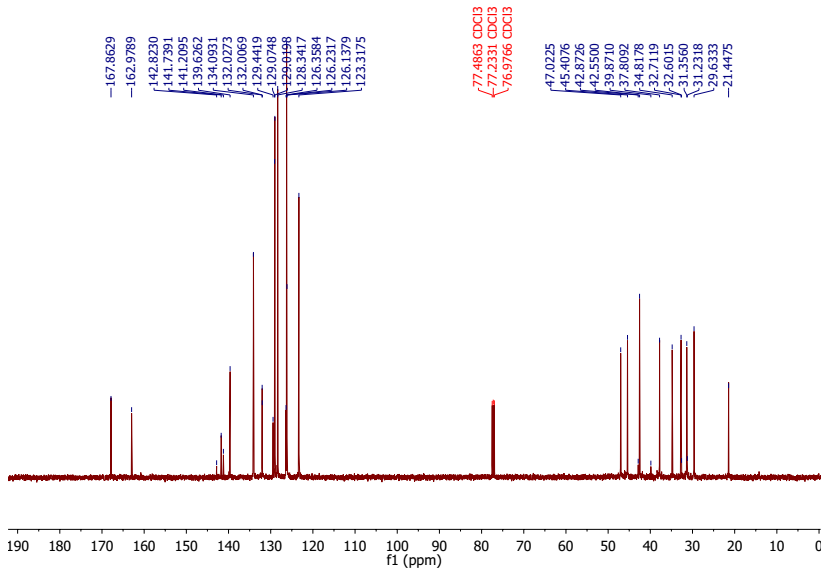
*

*N-(3-(1,3-dioxoisoindolin-2-yl)-1-(4-(4-methoxybenzyl)piperidin-1-yl)propylidene)benzenesulfonamide* ***4k****
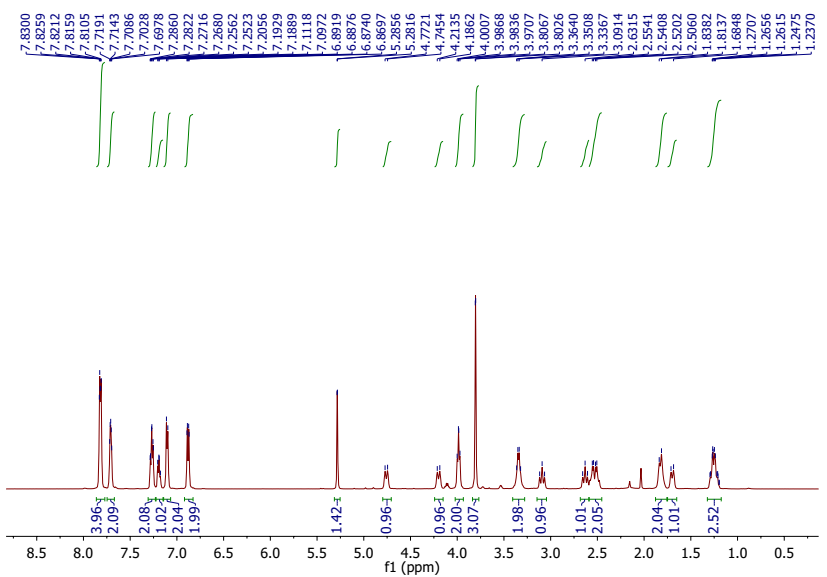

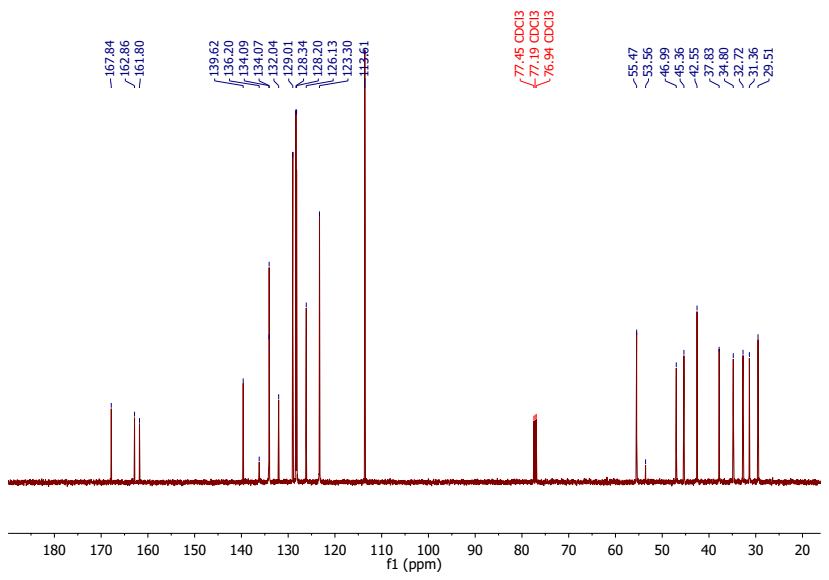
*

*N-(3-(1,3-dioxoisoindolin-2-yl)-1-(4-phenylpiperazin-1-yl)propylidene)benzenesulfonamide* ***4l***

*
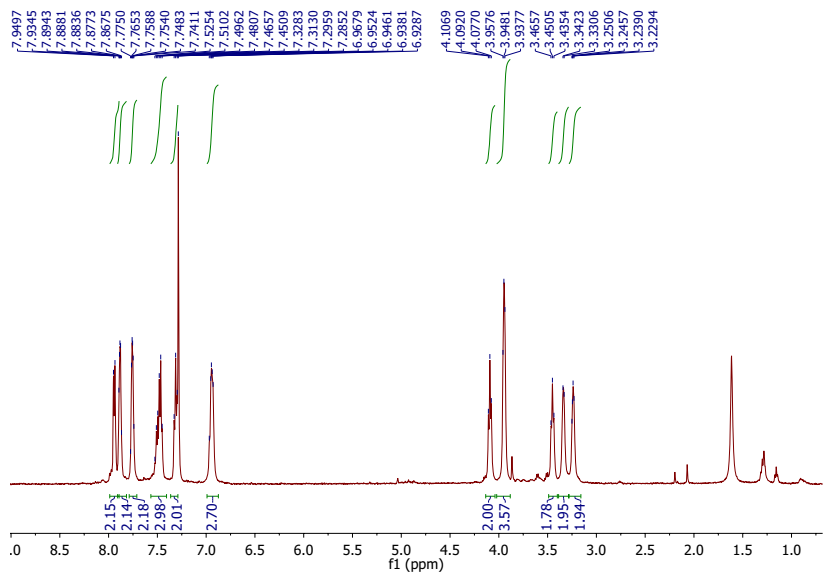
*

*
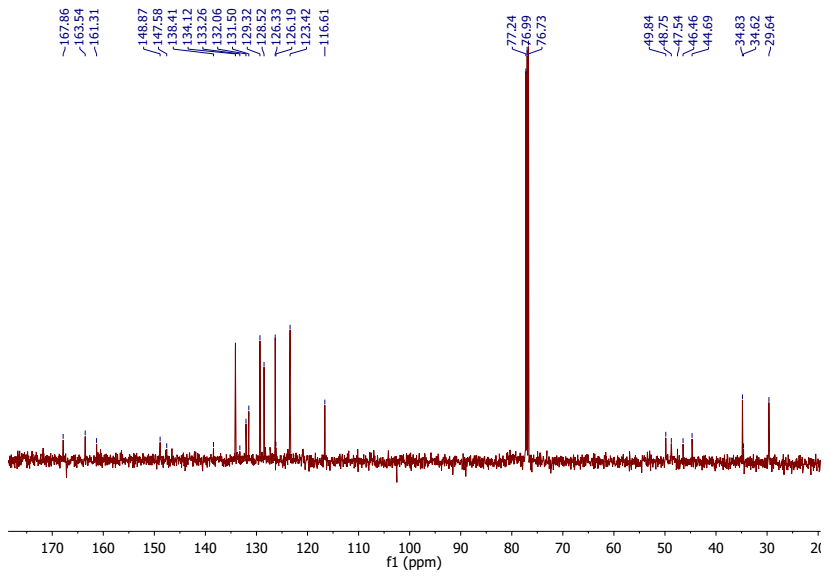
*

* N-(3-(1,3-dioxoisoindolin-2-yl)-1-(4-(p-tolyl)piperazin-1-yl)propylidene)benzenesulfonamide* ***4m***

*
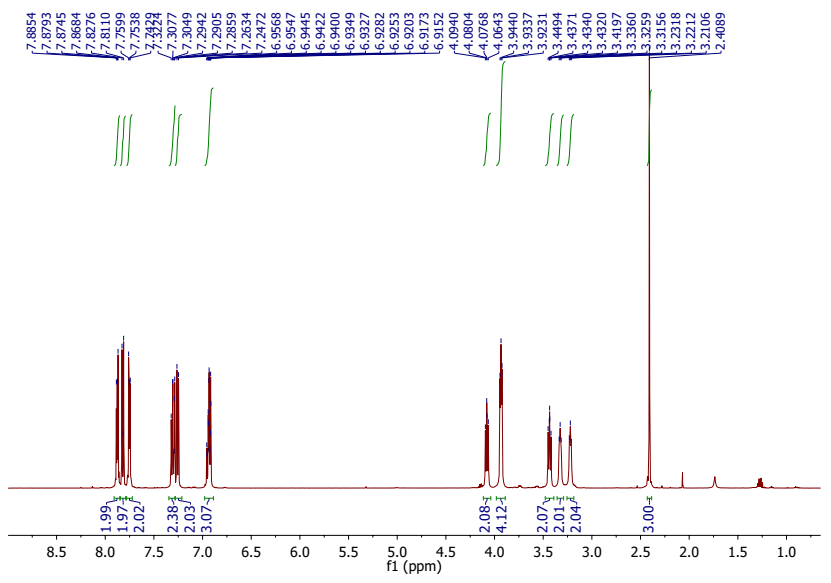
*

*
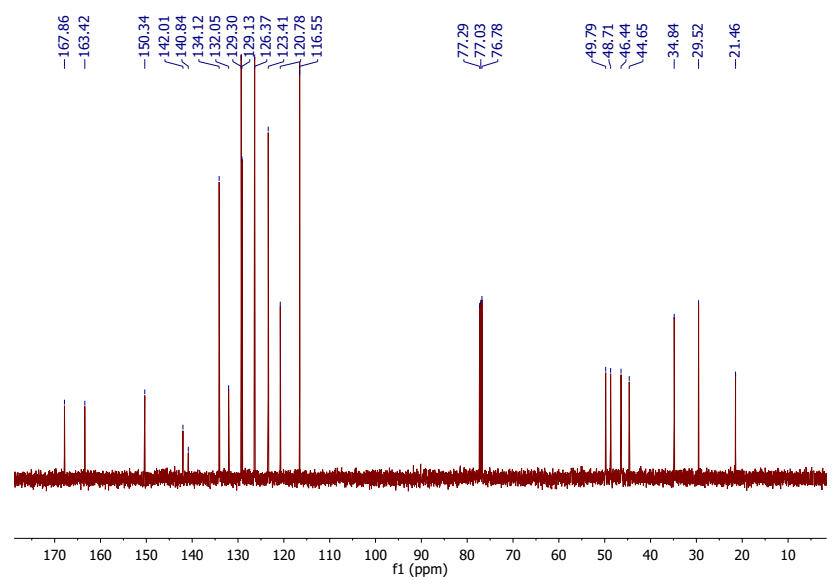
*

* N-(3-(1,3-dioxoisoindolin-2-yl)-1-(4-(4-methoxyphenyl)piperazin-1-yl)propylidene)benzenesulfonamide* ***4n***

*
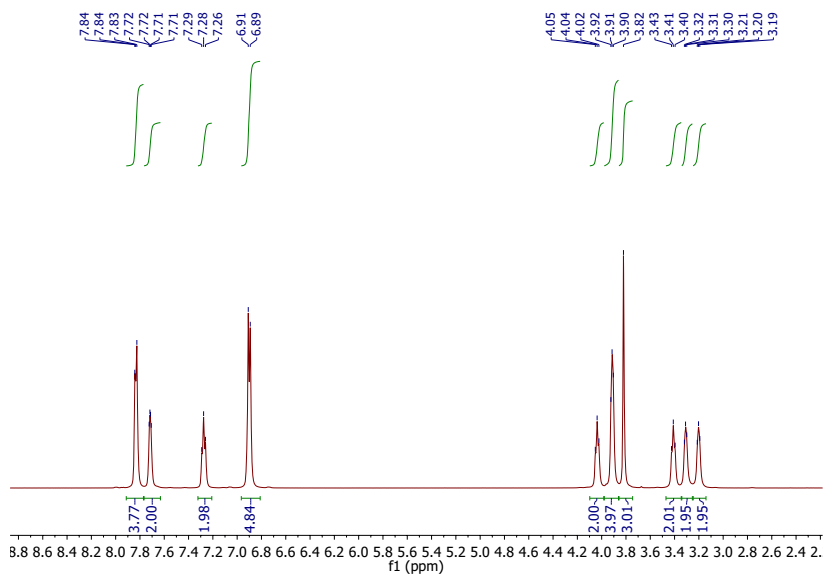
*

*
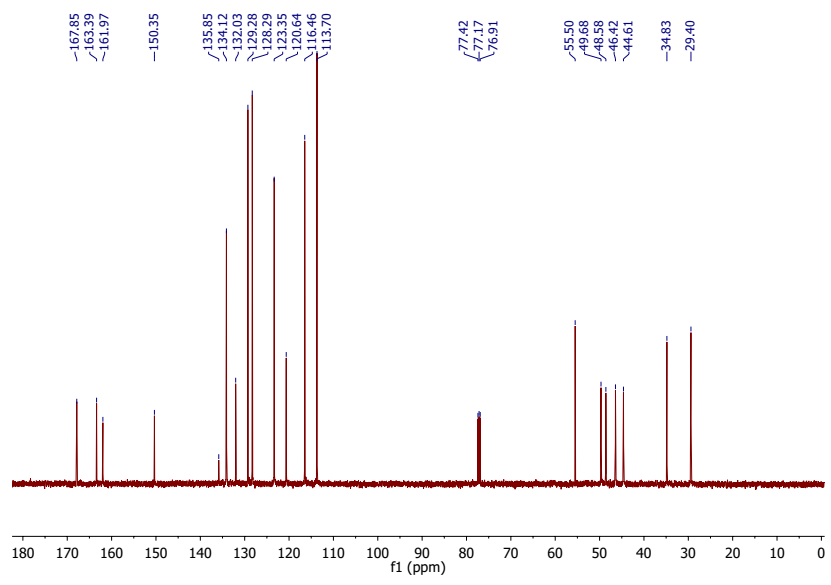
*
